# The GbsR Family of Transcriptional Regulators: Functional Characterization of the OpuAR Repressor

**DOI:** 10.3389/fmicb.2018.02536

**Published:** 2018-10-24

**Authors:** Stefanie Ronzheimer, Bianca Warmbold, Christian Arnhold, Erhard Bremer

**Affiliations:** ^1^Laboratory for Microbiology, Department of Biology, Philipps-Universität Marburg, Marburg, Germany; ^2^LOEWE Center for Synthetic Microbiology, Philipps-Universität Marburg, Marburg, Germany

**Keywords:** MarR, repressor, ABC transporters, osmotic stress, compatible solutes, glycine betaine, choline

## Abstract

Accumulation of compatible solutes is a common stress response of microorganisms challenged by high osmolarity; it can be achieved either through synthesis or import. These processes have been intensively studied in *Bacillus subtilis*, where systems for the production of the compatible solutes proline and glycine betaine have been identified, and in which five transporters for osmostress protectants (Opu) have been characterized. Glycine betaine synthesis relies on the import of choline via the substrate-restricted OpuB system and the promiscuous OpuC transporter and its subsequent oxidation by the GbsAB enzymes. Transcription of the *opuB* and *gbsAB* operons is under control of the MarR-type regulator GbsR, which acts as an intracellular choline-responsive repressor. Modeling studies using the X-ray structure of the Mj223 protein from *Methanocaldococcus jannaschii* as the template suggest that GbsR is a homo-dimer with an N-terminal DNA-reading head and C-terminal dimerization domain; a flexible linker connects these two domains. In the vicinity of the linker region, an aromatic cage is predicted as the inducer-binding site, whose envisioned architecture resembles that present in choline and glycine betaine substrate-binding proteins of ABC transporters. We used bioinformatics to assess the phylogenomics of GbsR-type proteins and found that they are widely distributed among *Bacteria* and *Archaea*. Alignments of GbsR proteins and analysis of the genetic context of the corresponding structural genes allowed their assignment into four sub-groups. In one of these sub-groups of GbsR-type proteins, *gbsR*-type genes are associated either with OpuA-, OpuB-, or OpuC-type osmostress protectants uptake systems. We focus here on GbsR-type proteins, named OpuAR by us, that control the expression of *opuA*-type gene clusters. Using such a system from the marine bacterium *Bacillus infantis*, we show that OpuAR acts as a repressor of *opuA* transcription, where several compatible solutes (e.g., choline, glycine betaine, proline betaine) serve as its inducers. Site-directed mutagenesis studies allowed a rational improvement of the putative inducer-binding site in OpuAR with respect to the affinity of choline and glycine betaine binding. Collectively, our data characterize GbsR-/OpuAR-type proteins as an extended sub-group within the MarR-superfamily of transcriptional regulators and identify a novel type of substrate-inducible import system for osmostress protectants.

## Introduction

Many microorganisms are confronted in their natural habitats either with permanent or temporary high osmolarity or high salinity surroundings (Galinski and Trüper, 1994; Ventosa et al., 1998). Such environments impose a considerable strain on the bacterial cell because the resulting difference in the osmotic potential between the cells’ cytoplasm and that of the exterior inevitably triggers water efflux. The ensuing dehydration of the cytoplasm, the drop in turgor to non-physiological values, and the increase in molecular crowding strongly affect growth and survival of the bacterial cell (Bremer and Krämer, 2000; Wood, 2011; van den Berg et al., 2017). To cope with cellular stress elicited by high osmolarity environments, many bacteria use the accumulation of compatible solutes as a common strategy (Csonka, 1989; Kempf and Bremer, 1998; Wood et al., 2001; Sleator and Hill, 2002). Compatible solutes, biochemically and physiologically compliant organic osmolytes (Cayley et al., 1992; Ignatova and Gierasch, 2006; Street et al., 2006; Wood, 2011; Stadmiller et al., 2017), can be amassed by microorganisms under osmotic stress conditions either through synthesis or uptake (Bremer and Krämer, 2000). For energetic reasons, the import of pre-formed osmostress protectants is preferred over their *de novo* synthesis or their production from imported precursor molecules (Oren, 1999).

Uptake and synthesis of compatible solutes (e.g., L-proline and glycine betaine) is particularly well studied in *Bacillus subtilis*. This soil bacterium possesses osmostress-responsive biosynthetic pathways for the compatible solutes L-proline and glycine betaine and harbors five osmotically inducible uptake systems (Opu) for a large number of osmostress protectants (Hoffmann and Bremer, 2016, 2017). Osmostress-responsive proline biosynthesis occurs *de novo* (Whatmore et al., 1990; Brill et al., 2011), but the production of glycine betaine requires the prior import of the precursor choline (Boch et al., 1994, 1996). Choline uptake is mediated via the substrate-restricted OpuB and the broad-substrate-accepting OpuC ABC-type transporters (Kappes et al., 1999; Teichmann et al., 2017) and is subsequently oxidized to glycine betaine by the GbsB and GbsA enzymes (Boch et al., 1996). The choline-responsive GbsR regulatory protein coordinates the expression of the *gbsAB* and *opuB* operons but this repressor does not regulate the transcription of the *opuC* gene cluster encoding the promiscuous OpuC transporter (Nau-Wagner et al., 2012; Hoffmann and Bremer, 2017; Teichmann et al., 2017).

GbsR is a member of the superfamily of MarR-type regulators (Nau-Wagner et al., 2012). These types of transcription factors control the expression of genes with various physiological functions, including metabolic pathways, virulence genes, and determinants for multi-drug resistance (Deochand and Grove, 2017; Grove, 2017). MarR-type proteins possess a common structural fold where the N-terminal DNA reading head contains a winged helix-turn-helix motif and where the C-terminal domain facilitates dimerization and inducer binding (Deochand and Grove, 2017; Grove, 2017).

An *in silico* model for the *B. subtilis* GbsR protein has previously been developed using the crystal structure of the *Methanococcus* (*Methanocaldococcus*) *jannaschii* Mj223 protein as the template (Nau-Wagner et al., 2012). In analyzing the Mj223 crystal structure (Ray et al., 2003), a regulator that has been suggested to play a role in the genetic control of multi-drug resistance determinant(s), Ray et al. (2003) noticed that the two DNA-reading heads in the Mj223 dimer assembly would not fit onto a standard B-form of DNA to interact with the presumed operator site(s). Modeling studies conducted by these authors suggest that substantial spatial rearrangements of both the DNA-binding and dimerization domains are required to allow an interaction of the Mj223 protein with DNA. These envisioned movements pivot around a flexible linker region connecting the DNA-binding and dimerization domains of the Mj223 protein (Ray et al., 2003).

**FIGURE 1:**
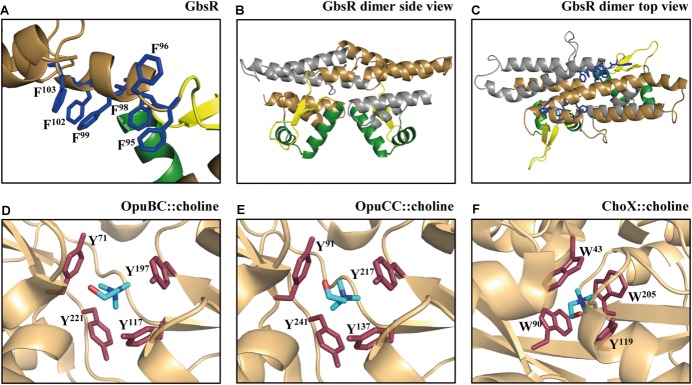
Structural comparison of the choline-binding pockets present in the OpuBC, OpuCC, ChoX substrate-binding proteins with the *in silico* predicted inducer-binding site of the *B. subtilis* GbsR repressor. **(A)** *In silico* model of the monomer of the *B. subtilis* GbsR protein (Nau-Wagner et al., 2012); it is based on the crystal structure of the DNA-binding protein Mj223 of *M. jannaschii* (PDB entry 1KU9) (Ray et al., 2003). **(B,C)** A side and top view, respectively, of the *in silico* predicted GbsR dimer. The winged helix-turn-helix region is represented in green, the inter-domain linker region is shown in yellow, and the six aromatic amino acids potentially involved in structuring the inducer-binding site are highlighted in blue. Crystal structures of **(D)** the *B. subtilis* OpuBC protein (PDB entry 3R6U; Pittelkow et al., 2011), **(E)** the *B. subtilis* OpuCC protein (PDB entry 9PPQ; Du et al., 2011), and **(F)** the *Sinorhizobium meliloti* ChoX protein (PDB entry 2REG; Oswald et al., 2008) all in complex with a choline molecule (blue sticks) are depicted. Aromatic amino acids involved in choline binding are highlighted in red.

By inspecting the Mj223-derived *in silico* model of the *B. subtilis* GbsR protein, we noticed a striking clustering of aromatic amino acids whose side-chains could potentially form an aromatic cage-like structure that is positioned near the flexible linker region (Nau-Wagner et al., 2012; Figures 1A–C). Aromatic cage-like structures are characteristic features of many substrate-binding proteins mediating the high-affinity capturing of osmostress protectants with fully methylated head-groups for their import into the cytoplasm via ABC transport systems (Schiefner et al., 2004a,b; Horn et al., 2006; Oswald et al., 2008; Smits et al., 2008; Wolters et al., 2010; Du et al., 2011; Pittelkow et al., 2011; Lang et al., 2015; Teichmann et al., 2018). In these aromatic cages, the positively charged head-group of the substrate (e.g., choline, glycine betaine, proline betaine) is coordinated via cation–π interactions (Schiefner et al., 2004a,b; Mahadevi and Sastry, 2013). Examples of these aromatic cages observed in crystal structures of substrate-binding proteins in complex with choline (OpuBC, OpuCC, ChoX) are shown in Figures 1D–F (Oswald et al., 2008; Du et al., 2011; Pittelkow et al., 2011). The presence of choline in the growth medium triggers enhanced expression of the *B. subtilis gbsAB* and *opuB* operons and purified GbsR binds choline *in vitro* with a *K*_d_ value of 165 ± 15 μM (Nau-Wagner et al., 2012). Building on what has been learned from the crystallographic analysis of choline-binding proteins (Oswald et al., 2008; Du et al., 2011; Pittelkow et al., 2011), the aromatic cage-like structure observed in the *in silico* model of GbsR (Figures 1A,C) is the prime candidate for the inducer-binding site. Since the corresponding region is absent in the *M. jannaschii* Mj223 template protein (Ray et al., 2003) used to generate the GbsR *in silico* model (Nau-Wagner et al., 2012), the spatial orientation of the side chains of the six aromatic residues that could potentially form such an aromatic cage in the GbsR repressor protein (Figure 1A) is unlikely to be correct. However, the overall fold of the dimeric GbsR protein predicted by the *in silico* model (Figures 1B,C) closely reflects the common structure observed in MarR-type regulators (Deochand and Grove, 2017; Grove, 2017).

In addition to GbsR, two other GbsR-related proteins [YvaV and OpcR (YvbF)] are present in *B. subtilis*; they exhibit an amino acid sequence identity of 34 and 35%, respectively, to the GbsR protein (Nau-Wagner et al., 2012). Their structural genes are part of a duplicated chromosomal region (Barbe et al., 2009) that also comprises the operons encoding the closely related OpuB and OpuC ABC transporters (Kappes et al., 1999; Teichmann et al., 2017). *yvaV* is positioned next to *opuB*, while *opcR* (*yvbF*) is located next to *opuC* (Barbe et al., 2009; Nau-Wagner et al., 2012). OpcR serves as a repressor for both the *opuB* and *opuC* operons (Lee et al., 2013) but no regulatory function has so far been ascribed to the YvaV protein. Neither of these proteins is involved in controlling the transcription of the *gbsAB* glycine betaine synthesis genes (Nau-Wagner et al., 2012). Like GbsR, the YvaV and OpcR proteins seem to possess aromatic-cage-like effector binding sites, but in contrast to GbsR (Nau-Wagner et al., 2012), no ligand has been identified that would affect the DNA-binding of these two GbsR-type proteins.

The fact that three *gbsR*-type genes exist in *B. subtilis* that are associated with genes for cellular osmotic stress response systems kindled our interest in this type of regulatory protein. In this report, we now explore the phylogenomics of GbsR-type proteins in both *Bacteria* and *Archaea*. This *in silico* analysis revealed that GbsR-related proteins form a substantial subgroup within the MarR-family of transcription factors (Deochand and Grove, 2017; Grove, 2017) and that they are not only associated with osmostress response genes. In our analysis, we paid particular attention to *gbsR*-type genes located in the vicinity of genes encoding putative glycine betaine synthesis enzymes or compatible solute uptake systems. Using the marine isolate *Bacillus infantis* as an example, we describe here functional studies with the GbsR-type OpuAR regulatory protein that is associated with an OpuA-type (Kempf and Bremer, 1995) compatible solute ABC importer. OpuAR was found to act as a repressor of *opuA* transcription and its DNA-binding activity reacts *in vivo* to several compatible solutes, including those that serve as substrates for the *B. infantis* OpuA transporter. Through site-directed mutagenesis rationally targeting residues forming the predicted aromatic cage, we were able to significantly improve the affinity of the OpuAR repressor protein for its effector molecules choline and glycine betaine, thereby supporting the envisioned role of this structure as the inducer-binding site.

## Materials and Methods

### Chemicals

The chromogenic substrate for the TreA reporter enzyme assays, *para*-nitrophenyl-α-D-glucopyranoside (α-PNPG), was purchased from Sigma-Aldrich (Steinheim, Germany). Antibiotics were acquired from Carl Roth (Karlsruhe, Germany), United States Biochemical Corp. (Cleveland, OH, United States), Sigma-Aldrich (Steinheim, Germany), and InvivoGen (San Diego, CA, United States). Anhydrotetracycline hydrochloride (AHT), Strep-Tactin Superflow chromatography material, and desthiobiotin were purchased from IBA (Göttingen, Germany). The used compatible solutes were all from laboratory stocks; their origins have been previously detailed (Hoffmann and Bremer, 2011).

### Bacterial Strains

The *B. infantis* strain NRRL B-14911 (Siefert et al., 2000) (BGSC Accession Code: 29A3) was obtained from the Bacillus Genetic Stock Center (BGSC) (Columbus, OH, United States). All *B. subtilis* strains used in this study are derivatives of the domesticated laboratory strain JH642 (Smith et al., 2014), and their genotypes are listed in Supplementary Table S1. The *Escherichia coli* K-12 strain DH5α (Invitrogen, Carlsbad, CA, United States) was used for routine cloning of plasmids and their maintenance. The *E. coli* B strain BL21 (Dubendorff and Studier, 1991) was used for overproduction of the recombinant *B. infantis* OpuAR protein and its mutant derivatives.

### Media and Growth Conditions

Bacterial strains were propagated in Luria–Bertani (LB) liquid media at 37°C or plated on LB agar plates. The *B. infantis* strain NRRL B-14911 was grown in MOPS-buffered basal medium [50 mM MOPS (pH 7.5), 50 mM MgSO_4_, 10 mM KCl, 10 mM CaCl_2_, 190 mM NH_4_Cl, 0.33 mM K_2_HPO_4_, 0.1 mM FeSO_4_] supplemented with 0.5% glucose (wt/vol) as a carbon source, 0.4% casamino acids (wt/vol), 5 ml l^-1^ vitamin solution A (7.8 mg l^-1^ biotin, 15.6 mg l^-1^ nicotinic acid, and 15.6 mg l^-1^ lipoic acid; pH adjusted to 7.5), and 5 ml l^-1^ vitamin solution B (15.6 mg l^-1^ pantothenic acid, 15.6 mg l^-1^ pyridoxine-HCl, 15.6 mg l^-1^ thiamine, 15.6 mg l^-1^ *p*-aminobenzoic acid, and 0.32 mg l^-1^ cobalamin) (Gonzalez et al., 1997). In main cultures used for osmostress protection assays, casamino acids were left out from the growth medium. The osmolarity of growth media was adjusted by the addition of NaCl from a 5 M NaCl stock solution. If desired, osmostress protectants were added to the medium of *B. infantis* NRRL B-14911 cultures at a final concentration of 1 mM. For growth experiments, liquid cultures of *B. infantis* NRRL B-14911 were grown in 100-ml Erlenmeyer flasks containing 20-ml of medium in a water bath set to 37°C with vigorous shaking (set to 220 rpm). Pre-cultures of strains were propagated to mid-exponential growth phase in basal medium (with 0.4% casamino acids) and were then used to inoculate fresh basal medium (without casamino acids) to an optical density at 578 nm (OD_578_) of 0.1. Liquid cultures of *B. subtilis* strains were grown at 37°C in Spizizen’s minimal medium (SMM) (Harwood and Archibald, 1990) with 0.5% glucose as carbon source and a solution of trace elements (Harwood and Cutting, 1990). L-Tryptophan (20 mg l^-1^) and L-phenylalanine (18 mg l^-1^) were added to SMM-derived growth media to satisfy the auxotrophic needs of the *B. subtilis* strain JH642 (*trpC2 pheA1*) (Smith et al., 2014) and its derivatives (Supplementary Table S1). Cultures (20-ml in 100-ml Erlenmeyer flasks) of *B. subtilis* strains were inoculated from exponentially growing pre-cultures in SMM to an OD_578_ of 0.1 and were incubated in a shaking water bath (set to 220 rpm) at 37°C. If needed, the osmolarity of the growth medium was increased by the addition of NaCl (from 5 M stock solutions prepared in H_2_O). Compatible solutes were filter sterilized and added to the growth medium of *B. subtilis* strains from 100 mM stock solutions to a final concentration of 1 mM unless otherwise explicitly stated in the text.

### Cloning and Site-Directed Mutagenesis of the *opuAR* Gene From *B. infantis* NRRL B-14911

The coding region of *opuAR* was amplified from chromosomal DNA of *B. infantis* NRRL B-14911 (Massilamany et al., 2016) using primers opuAR fwd and opuAR rev (Supplementary Table S2) harboring in their 5′-regions recognition sites for the restriction enzyme BsaI. The resulting PCR DNA fragment was then cut with BsaI and cloned into the expression vector pASK-IBA3plus (IBA, Göttingen, Germany) that had been linearized with BsaI; the obtained plasmids were named pMP_AR1 (Supplementary Table S3). Mutant derivatives of the *opuAR* gene were obtained using the Q5 Site-Directed Mutagenesis Kit (New England BioLabs, Ipswich, MA, United States) and a set of customized mutagenic primers (Supplementary Table S2). The Sanger sequencing method was used to verify the DNA sequence of the chromosomal insert of the parental *opuAR*^+^ overexpression plasmid pMP_AR1 and of its derivatives carrying mutant *opuAR* genes. DNA sequencing was conducted by Microsynth (Lindau, Germany).

### Construction of *B. subtilis* Strains

For the heterologous expression of the *B. infantis* NRRL B-14911 *opuA* operon (*opuAA-opuAB-opuAC*) in *B. subtilis*, a 3826-bp DNA fragment including the coding region of the *opuA* operon and the adjacent *opuAR* regulatory gene was generated by PCR using primers CA3-opuARA fwd and CA3-opuARA rev (Supplementary Table S2). The PCR product (cleaved with BamHI) was cloned into the vector pX (Kim et al., 1996) that had been cut with BamHI; this resulted in plasmid pCA-*opuARA* (Supplementary Table S3). Plasmid pX and its recombinant derivatives allow the stable insertion of genes into the *B. subtilis* chromosome as a single copy into the non-essential *amyE* locus via a double homologous recombination event via *amyE* sequences present on both the plasmid and the chromosome (Kim et al., 1996). Insertion of plasmid pX, or its recombinant derivatives, into the *B. subtilis* chromosome at the *amyE* gene can be selected for on agar plates containing the antibiotic chloramphenicol (final concentration: 5 μg ml^-1^) and the subsequently scoring of the AmyE negative phenotype of the strains on LB plates containing 1% starch (Harwood and Cutting, 1990). Linearized plasmid DNA of pCA-*opuARA* was used to transform the *B. subtilis* chassis strain TMB118 [*Δ(opuA::tet)3 Δ(opuC::spc)3 Δ(opuD::neo)2 Δ(opuB::erm)3*] (Teichmann et al., 2017) in which only the proline-specific osmostress OpuE transporter (von Blohn et al., 1997) is present. The resulting strain was CAB2 (Supplementary Table S1). *B. subtilis* mutants carrying chromosomal deletions of the *yvaV* and *opcR* genes were constructed using long-flanking region PCR (Kuwayama et al., 2002). Primers used for the amplification of the 5′- and the 3′-regions flanking the gene of interest and the antibiotic resistance cassettes used to disrupt the coding region are listed in Supplementary Table S2. The tetracycline resistance cassette inserted in the *yvaV* locus was amplified using plasmid pDG1515 (Guerout-Fleury et al., 1995) as the template, and the zeocin resistance cassette inserted in the *opcR* gene was derived from plasmid p7Z6 (Yan et al., 2008). Fusion PCR products containing the 5′-flanking region, the antibiotic resistance cassettes, and the 3′-flanking region were used to transform derivatives of *B. subtilis* JH642, thereby yielding strains which are lacking all three GbsR-like regulators or strains which contained only one of them (Supplementary Table S1).

opuAA_B.infantis_′*-treA* reporter gene fusions were constructed using primers opuAR treA Frag1/2 rev and either opuAR treA Frag1/4 for (Supplementary Table S2) to amplify a 1940-bp DNA fragment from chromosomal DNA of *B. infantis* NRRL B-14911, including the predicted *opuA* promoter region and the coding region of *opuAR*, or primer opuAR treA Frag2 for, to generate a fragment lacking the *opuAR* gene. PCR products, which had been cut with BamHI and SmaI, were cloned into the vector pJMB1 (Hoffmann et al., 2013). This vector carries a promoterless *treA* gene, whose gene product [phospho-α-(1,1)-glucosidase] can be assayed and photometrically quantitated with the chromogenic substrate α-PNPG (Gotsche and Dahl, 1995). The plasmids resulting for the construction of the *treA* transcriptional reporter gene fusions were named pSTH33 (*opuAR^+^*/*opuAA_B.i_*-*treA*) and pSTH34 (*opuAR^-^*/*opuAA_B.i_*-*treA*) (Supplementary Table S3). All *treA* reporter gene fusions were stably integrated as a single copy into the chromosome of various *B. subtilis* strains at the *amyE* gene; all of these strains carry a gene disruption mutation of the native chromosomal *treA* gene of *B. subtilis* to allow the assignment of the measured TreA enzyme activity to the reporter gene constructs (Supplementary Table S1).

### TreA Enzyme Activity Assays

Aliquots (1.5 ml) from cultures of *B. subtilis* strains carrying chromosomal opuAA__B.i__′*-treA* reporter operon gene fusions (Supplementary Table S1) were used to monitor the expression levels by assaying the TreA reporter enzyme activity as described previously using the chromogenic substrate α-PNPG (Gotsche and Dahl, 1995). The TreA-specific activity is expressed in units per milligram of protein. Protein concentrations were estimated from the optical density of the cell culture harboring the *treA* reporter operon gene fusion (Miller, 1972).

### Overproduction and Purification of Recombinant OpuAR Proteins

Overproduction of the OpuAR repressor protein and its mutant derivatives was carried out in the *E. coli* B strain BL21 harboring plasmid pMP_AR1, a derivative of the expression vector pASK-IBA3plus (IBA, Göttingen, Germany) (Supplementary Table S3). In pMP_AR1, the 3′-end of the *B. infantis opuAR* coding region is fused to a short DNA fragment encoding a *Strep*-tag II affinity peptide (SA-WSHPQFEK). In this plasmid, the *opuAR* gene is expressed from the P-*tet* promoter, whose transcriptional activity is under control of the TetR repressor whose structural gene is present in the backbone of the expression vector. De-repression of P-*tet* promoter activity is achieved by adding the synthetic inducer AHT to the growth medium. Cultures of the *E. coli* B. strain BL21 (pMP_AR1) were inoculated (to a OD_578_ of 0.1) from pre-cultures prepared in MMA (Miller, 1972) supplemented with 0.5% glucose (wt/vol) as the carbon source, 0.5% casamino acids (wt/vol), 1 mg l^-1^ thiamine, and 1 mM MgSO_4_. The cultures also contained ampicillin (100 μg ml^-1^) to select for the presence of plasmid pMP_AR1 (or its mutant derivatives) and they were grown at 37°C. When the main cultures reached an OD_578_ of 0.5, enhanced expression of the *opuAR* gene from P-*tet* was induced by the addition of AHT to a final concentration of 0.2-μg ml^-1^. Cells were grown for an additional 2 h, before they were harvested by centrifugation (at 4°C at 4,800 × *g* for 20 min). The cell pellets were re-suspended in lysis buffer [100 mM Tris-HCl (pH 7.5), 2.5% glycerol, 2 mM dithiothreitol, 0.4 mM EDTA, 0.5 mM phenylmethylsulfonyl fluoride (PMSF), 0.5 mM benzamidine] and the cells were then disrupted by passing them through a French pressure cell as detailed previously (Nau-Wagner et al., 2012). Cleared cell lysates were prepared by ultra-centrifugation (at 4°C at 100,000 × g for 35 min) before the OpuAR-*Strep*-tag II recombinant protein was purified by affinity chromatography (Nau-Wagner et al., 2012) on streptactin affinity resin according to the manufacturers specifications (IBA, Göttingen, Germany). Mutant derivatives of the OpuAR protein were overproduced and purified as described above for the wild-type protein.

### Determination of the Quaternary Assembly of the Purified OpuAR Protein

To analyze the quaternary assembly of the OpuAR protein of *B. infantis*, we performed size-exclusion chromatography. For these experiments, the overproduction of the OpuAR repressor protein was carried out as described above but the buffer for its affinity-purification was changed [100 mM KPP (pH 8) supplemented with 300 mM NaCl] to improve OpuAR protein stability. Immediately after purification, 2-ml protein solution (1.5 mg ml^-1^) was loaded onto a size-exclusion chromatography column (HiLoad 16/600 Superdex 200 pg; GE Healthcare, Münschen, Germany) that was run in a buffer containing 100 mM KPP (pH 8) and 300 mM NaCl. The following proteins were used to standardize the size-exclusion chromatography column: thyroglobulin (667 kDa), albumin (66 kDa), ovalbumin (43 kDa), and cytochrome C (12.4 kDa). These marker proteins were purchased from GE Healthcare (München, Germany) and from Sigma-Aldrich (Steinheim, Germany). The purity and molecular mass of the OpuAR protein subsequent to size-exclusion chromatography was assessed by SDS-polyacrylamide gel electrophoresis (15%); proteins were stained with Coomassie Brilliant Blue.

### Determination of the Dissociation Constant of the OpuAR::Choline and OpuAR::Glycine Betaine Complexes

The OpuAR protein, purified by affinity chromatography, was concentrated using VivaSpin 6 columns (Sartorius AG, Göttingen, Germany) with a simultaneous change from the purification buffer [100 mM Tris–HCl (pH 7.5) 150 mM NaCl] to a solution containing 25 mM Tris–HCl (pH 7.5), 25 mM NaCl. The dissociation constants of OpuAR for choline and glycine betaine were determined by intrinsic tryptophan fluorescence spectroscopy as described previously using a Carry Eclips fluorescence spectrometer (Varian, Surry, United Kingdom) (Pittelkow et al., 2011; Nau-Wagner et al., 2012). The excitation wavelength of the fluorescence spectrometer was set to 280 nm, the slit width was 5 nm, and the photomultiplier tube voltage (PMT) of the fluorescence detector was set to 800 V; the emission spectrum of the recombinant OpuAR protein was recorded in a range between 290 and 400 nm. Purified OpuAR protein (5 μM) was titrated with various concentrations of either choline (25–1,600 μM) or glycine betaine (25–1,300 μM), and the differences in the intrinsic fluorescence intensity, caused by ligand binding by the OpuAR protein, were used to calculate the apparent *K*_d_ (equilibrium dissociation constant) value (Pittelkow et al., 2011; Nau-Wagner et al., 2012).

### Bioinformatics

Genome sequences of members of the domains *Bacteria* and *Archaea* were retrieved from the IMG/M database accessible via the genome portal of the Department of Energy Joint Genome Institute (United States) (Chen et al., 2017). We restricted our database analysis to only one representative from each species/strain, and analyzed only those fully sequenced genomes for which 16S rDNA sequences deposited in the SILVA database (Glöckner et al., 2017) were also provided through the IMG/M database. Proteins homologous to the GbsR protein from *B. subtilis* JH642 (Nau-Wagner et al., 2012) were searched for using the BLAST-P algorithm (Altschul et al., 1990). The genome context in the immediate vicinity of *gbsR*-like genes was evaluated using the gene neighborhood tool^1^ provided by the IMG/M web-server. The amino acid sequences of GbsR homologs were aligned using the MAFFT web-server^2^ to analyze their phylogenetic relationship with standard bootstrap settings (100 bootstraps) automatically chosen by the web-server (Katoh et al., 2017). Based on a 16S rDNA alignment of strains harboring a GbsR homolog (final dataset: 146 entries), a phylogenetic tree was constructed to visualize the phylogenomic distribution of *gbsR*-type genes and the same dataset was also used to derive a GbsR protein homology tree; it was visualized using the Interactive Tree of Life (iTOL) web-tool^3^ (Letunic and Bork, 2016).

The amino acid sequences of the components of the *B. subtilis* OpuB and OpuC ABC transporters are closely related to each other because the *opuB* and *opuC* operon are likely the result of a gene duplication event (Kappes et al., 1999; Barbe et al., 2009; Teichmann et al., 2017). The least conserved component of these two transporter systems are their substrate binding proteins (OpuBC and OpuCC, respectively) with a degree of amino acid sequence identity of 71% of the mature proteins (Kappes et al., 1999). To assign a particular transporter to either the OpuB or OpuC family, an amino acid sequence alignment of the substrate-binding protein with either the OpuBC or OpuCC protein (Kappes et al., 1999) was performed. OpuA-type ABC transporters were identified by assessing the amino acid sequence relatedness with the OpuAC substrate-binding protein from *B. subtilis* (Kempf and Bremer, 1995). To identify OpuD and OpuE transporters in *B. infantis*, the corresponding proteins from *B. subtilis* (Kappes et al., 1996; von Blohn et al., 1997) were used for a BLAST-P search. To assess the presence of glycine betaine synthesis genes in *B. infantis*, the amino acid sequences of the *B. subtilis* GbsA and GbsB proteins (Boch et al., 1996) were used as the search templates. To search for the glycine betaine synthesis enzyme choline oxidase, we used the amino acid sequence of the CodA protein from *Arthrobacter globiformis* (Fan et al., 2004) as the search template.

*In silico* models of the GbsR protein of *B. subtilis* and the OpuAR protein from *B. infantis* were generated via the SWISS Model server^4^ (Waterhouse et al., 2018). For modeling of the GbsR and OpuAR proteins, crystallographic data of the MarR-type regulator Mj223 of *M. jannaschii* [Protein Data Bank (PDB) accession code: 1KU9] (Ray et al., 2003) were automatically used as the template by the SWISS Model server. Modeling of the GbsU proteins from *Halobacillus halophilus* and *Virgibacillus* sp. SK37 and those representing OpuAC-type substrate-binding proteins from *B. infantis* NRRL B-14911, and *Paenibacillus larvae* (DSM 25719) were also conducted with the SWISS Model server. Figures of protein structures were prepared using the PyMOL software package^5^ (Delano, 2002).

## Results

### Phylogenetic Distribution of GbsR-Type Regulators Among *Bacteria* and *Archaea*

**FIGURE 2:**
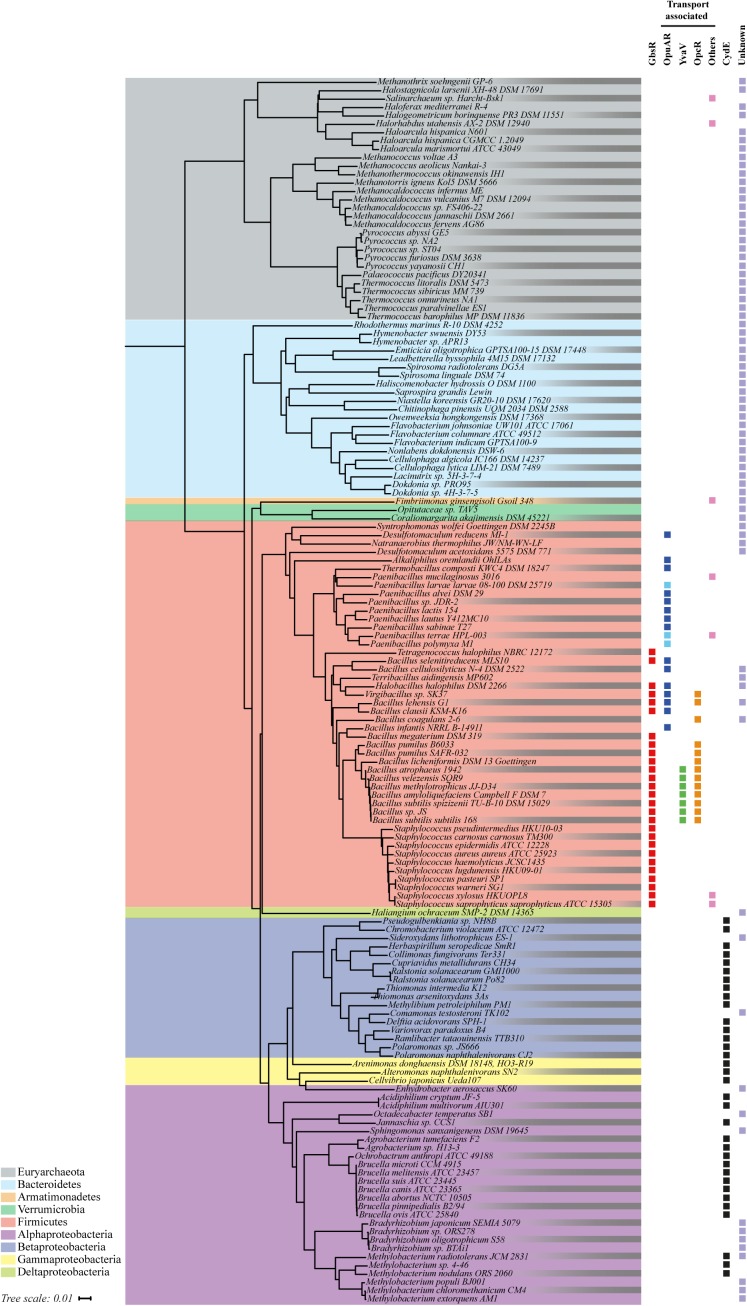
Phylogenetic distributions of GbsR-type proteins. 146 *Bacteria* and *Archaea* that harbor at least one copy of a *gbsR*-like gene were identified by bioinformatics. Information on fully sequenced microbial genomes were obtained from the IMG/M web-server, and homologs of the GbsR protein of *B. subtilis* JH642 (Nau-Wagner et al., 2012) were searched for via the JGI web-server (http://genome.jgi.doe.gov) (Chen et al., 2017) using the BLAST-P algorithm (Altschul et al., 1990). We restricted our analysis to only one representative from each species/strain and to those genome sequences for which 16S rDNA sequences were also available from the SILVA database (Glöckner et al., 2017). The phylogenetic tree was built based on a 16S rDNA alignment using tools provided by the JGI web-server. The retrieved GbsR homologs were grouped according to their immediate gene neighborhood. Those found in context with genes associated with glycine betaine synthesis were assigned as GbsR (red). *gbsR*-type genes encoded in the proximity of genes encoding transport systems for osmostress protectants were named OpuAR (blue), YvaV (green), or OpcR (orange), according to the adjacent genes encoding a particular type of Opu transporter. Pale blue boxes represent *gbsR* genes positioned next to *opuA* gene clusters containing a substrate-binding protein fused to the trans-membrane donain. Black boxes represent *gbsR*-like genes associated with genes encoding for cytochrome *bd*-type oxidases; these were named according to the proposal made by Xia et al. (2018) as CydE. If a transporter other than a Opu-type import system was found in the immediate vicinity of *gbsR*-type genes, these GbsR homologs were grouped as others (pink). The retrieved GbsR homologs were grouped as “unknown” if there was no assigned function for the neighboring gene(s) (pale purple).

We used the IMG/M database accessible via the genome portal of the Department of Energy Joint Genome Institute (Chen et al., 2017) and the amino acid sequence of the GbsR repressor protein from *B. subtilis* JH642 (Nau-Wagner et al., 2012) as the search query to assess the phylogenetic distribution of GbsR-like proteins among members of the *Bacteria* and *Archaea*. At the time of the BLAST-P search (6 May 2018), the database contained 172 fully sequenced archaeal and 3,523 bacterial genomes. We manually curated this dataset into a non-redundant group of genome sequences of microbial species and strains; it contained 150 genome sequences from *Archaea* and 1,650 genome sequences from *Bacteria*. We purposely excluded draft and permanent draft genome sequences from our analysis in order to unambiguously identify *gbsR*-type genes and those that are potentially functionally associated with it. Our database searches lead to a total number of 179 hits representing GbsR-type proteins from 146 microorganisms; 29 GbsR-type proteins originated from 29 *Archaea* and 150 GbsR-type proteins were derived from 117 representatives of the *Bacteria* (Figure 2). We then used the available 16s rDNA sequences from these 146 microorganisms to construct a phylogenetic tree in order to assess the phylogenomic distribution of microorganisms possessing *gbsR*-type genes (Figure 2).

All Archaea that contain GbsR-type proteins belong to the phylum of the *Euryarchaeota*, a group of highly diverse microorganisms that comprises different types of extremophilic and methanogenic representatives. In this group (Figure 2), *M. jannaschii* is found, a thermophilic representative of the Methanococci (Bult et al., 1996), whose crystalized Mj223 protein (Ray et al., 2003) had served as the template for the generation of the *in silico* model of the *B. subtilis* GbsR protein (Nau-Wagner et al., 2012). The other GbsR-containing microorganisms belong to the *Bacteria*, and in this dataset major groups belonging to the Alpha- and Betaproteobacteria, to the Firmicutes, and to the Bacteroidetes can be found (Figure 2).

As a next step in our *in silico* analysis of *gbsR*-type genes, we assessed the annotation of genes in their immediate neighborhood using the genome browser provided by the IMG/M web-server^6^. This allowed us to group the recovered GbsR-type proteins in four distinct classes. The genes for 73 of the identified GbsR homologs were present in immediate gene neighborhoods that did not allow us to deduce possible functions by the “guilty by the genetic association” approach (Rocha, 2008; Zhao et al., 2013) primarily belong to members of the Euryarchaeota and the Bacteroidetes (Figure 2). In contrast, the genes of the remaining 106 GbsR-type proteins were found in genomic neighborhoods that allowed a consistence binning into four classes: (i) a few (seven representatives) were found in the immediate vicinity of various types of transporter genes encoding possible importers for ammonium, molybdenum, sugars, and a glycine betaine uptake system of the BCCT-type transporter family (Ziegler et al., 2010). (ii) Thirty-four *gbsR*-type genes were found in immediate vicinity of *cydAB*-type or *cydABCD*-type operons, which encode oxygen reductase serving as an alternative terminal electron transfer step in the respiratory chain of many prokaryotes (Borisov et al., 2011; Degli Esposti et al., 2015; Xia et al., 2018). (iii) Twenty-seven *gbsR*-type genes were found in the immediate vicinity of glycine betaine synthesis gene clusters, a group of genes to which also the *B. subtilis gbsR* gene belongs (Nau-Wagner et al., 2012). (iv) Thirty-eight *gbsR*-type genes were located right next to gene clusters encoding putative OpuA-, OpuB-, and OpuC-type osmoprotectant uptake systems (Hoffmann and Bremer, 2016, 2017). The GbsR-type proteins found in this latter group comprises the OpcR and YvaV proteins from *B. subtilis* whose structural genes are divergently transcribed from the *opuC* and *opuB* operons, respectively (Nau-Wagner et al., 2012; Lee et al., 2013).

**FIGURE 3:**
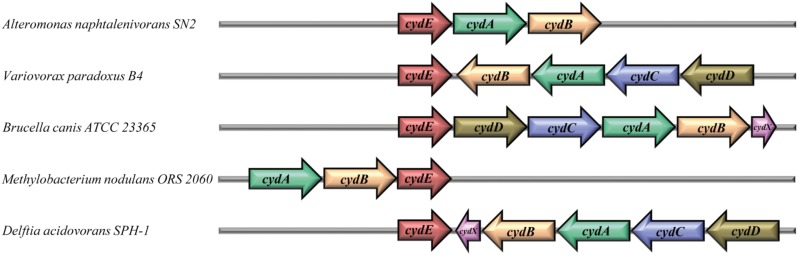
Gene-neighborhood of *gbsR*-like genes associated with genes encoding cytochrome *bd*-type oxygen reductases. Thirty-four genes for GbsR-like regulatory proteins [now addressed as CydE (Xia et al., 2018)] were identified in the immediate vicinity of gene clusters encoding an alternative terminal oxidase belonging to the cytochrome *bd*-family (Borisov et al., 2011; Degli Esposti et al., 2015). The *cydA* (green) and *cydB* (pale orange) genes encode the two subunits of the cytochrome *bd*-type oxygen reductase. The genes *cydC* (blue) and *cydD* (brown) encode an ABC-type transporter involved in the assembly of the cytochrome *bd* complex (Borisov et al., 2011; Degli Esposti et al., 2015). In some cases, *cydX* gene (pink) is located within the cytochrome *bd* oxidase operon, which was suggested to play a role in the assembly and stabilization of the di-heme center of CydAB oxidase (Hoeser et al., 2014; Chen et al., 2015).

The 34 *gbsR*-type genes found in the immediate vicinity of *cydAB*-type or *cydABCD*-type operons (Borisov et al., 2011; Degli Esposti et al., 2015) are present in genomes that all belong to members of the *Bacteria*; most of them are members of the Alpha- and Betaproteobacteria (Figure 2). In addition to their bioenergetics function, these types of oxygen reductases serve important physiological roles by facilitating the colonization of O_2_-poor ecological niches by both pathogenic and non-pathogenic bacteria, serve as O_2_-scavengers to protect oxygen-sensitive enzymes, and support anaerobic photosynthetic growth. They are particularly prevalent in the etiological agents causing brucellosis, tuberculosis, pneumonia, meningitis, and other types of severe infections (Borisov et al., 2011). The two subunits of the cytochrome *bd*-type oxygen reductase are encoded by the *cydA* and *cydB* genes. In some of the identified gene clusters, genes (*cydC* and *cydD*) are present that encode an ABC-type transporter that is involved in the assembly of cytochrome *bd* (Borisov et al., 2011). Furthermore, an additional gene, named *cydX*, is located within some of the cytochrome *bd* oxidase operons (Figure 3). It has been suggested that *cydX* plays a role in the assembly and stabilization of the di-heme center of the CydAB protein complex (Hoeser et al., 2014; Chen et al., 2015). Overall, the genetic configuration of *cyd* gene clusters observed by us (Figure 3) is widespread in proteobacterial genomes (Degli Esposti et al., 2015).

While our manuscript was under evaluation, Xia et al. (2018) reported their findings on the genetic regelation of the *cydAB* gene cluster from *Alishewanella* sp. WH16-1 and genome evaluations of closely related taxa (Xia et al., 2018). This particular *cydAB* gene cluster is crucial for chromate and sulfide resistance. These authors identified a regulatory gene (named *cydE* by these authors) in the immediate vicinity of the *Alishewanella* sp. WH16-1 *cydAB* operon (and of several related microbial taxa) that negatively controls *cydAB* transcription (Xia et al., 2018). Fully consistent with our findings (Figure 3), Xia et al. (2018) refer to the CydE protein as a GbsR-type regulator; we will follow the genetic nomenclature proposed by these authors.

We assessed the overall amino acid sequence identity of the 34 CydE regulatory proteins identified in our study (Figure 2) with that of the *B. subtilis* GbsR protein, the founding member of the GbsR family of transcriptional regulators (Nau-Wagner et al., 2012). It ranged between 21 (for the CydE protein from *Alteromonas naphthalenivorans* SN2) and 19% (for the CydE protein from *Methylobacterium* sp. 4-46) (Supplementary Figure S1).

*gbsR*-harboring microorganisms in which these genes are found in the immediate vicinity of biosynthetic genes for the osmostress protectant glycine betaine or transporters for the import of compatible solutes were of particular interest to our study. We identified 46 microorganisms with such a genetic configuration (Figure 2). All of these bacteria are members of the *Firmicutes*, with a dominant representation of the genera *Staphylococcus*, *Bacillus*, and *Paenibacillus* (Figure 2). We address in more detail in the following, first bacteria where *gbsR*-type genes are associated with glycine betaine synthesis genes and, subsequently, those microorganisms in which *gbsR*-type genes are associated with genes encoding osmostress protectant uptake systems.

### *In silico* Assessment of GbsR-Type Regulatory Genes Associated With Genes for Glycine Betaine Synthesis

Microorganisms can synthesize the trimethylammonium compound glycine betaine either through the sequential methylation of glycine (Nyyssölä et al., 2000), or through the oxidation of the precursor choline, a process that can be catalyzed by different type(s) of enzymes (Lamark et al., 1991; Boch et al., 1996; Salvi et al., 2014). In *Escherichia coli*, a membrane-bound choline dehydrogenase (BetA) catalyzes the conversion of choline into glycine betaine, with glycine betaine aldehyde as the intermediate. In this enzyme system, a separate glycine betaine aldehyde dehydrogenase (BetB) serves as a safeguard to prevent the accumulation of the chemically highly reactive glycine betaine aldehyde to cytotoxic levels (Lamark et al., 1991). In contrast, in *B. subtilis*, a type-III alcohol dehydrogenase (GbsB) catalyzes the initial oxidation of choline to glycine betaine aldehyde, which is then further oxidized to glycine betaine by the GbsA glycine betaine aldehyde dehydrogenase (Boch et al., 1996). In both organisms, the precursor choline needs to be imported, but different types of transport systems are used for this purpose. BetT, a member of the BCCT family (Ziegler et al., 2010), serves as the choline transporter in *E. coli* (Lamark et al., 1991) and OpuB/OpuC, members of the ABC transporter family (Davidson et al., 2008), perform this function in *B. subtilis* (Kappes et al., 1999). Two different types of choline-sensing regulatory proteins, BetI in *E. coli* (Lamark et al., 1991) and GbsR in *B. subtilis* (Nau-Wagner et al., 2012), control the expression of the choline import and glycine betaine biosynthetic genes in response to the availability of choline in the environment.

**FIGURE 4:**
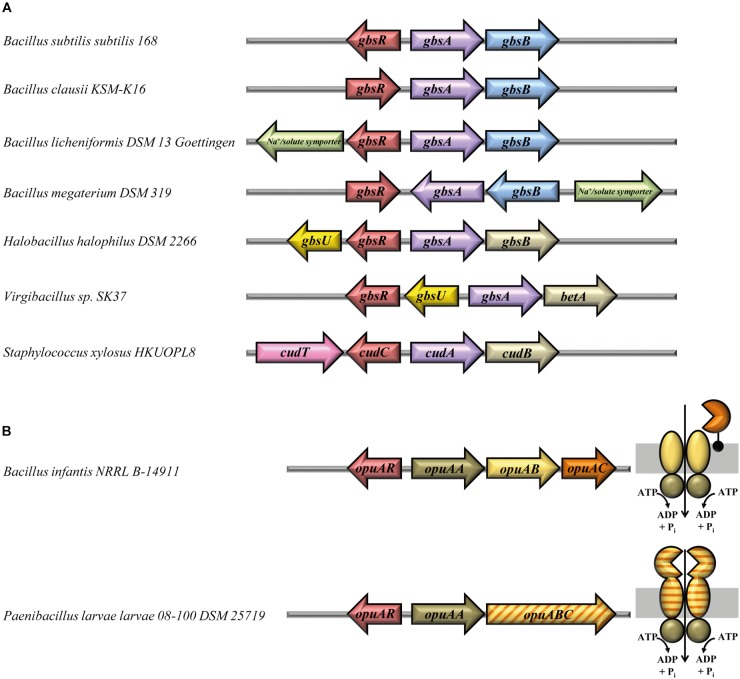
Gene-neighborhood of *gbsR*-like genes associated with glycine betaine synthesis and OpuA-type transporter genes. **(A)** Glycine betaine synthesis genes. Shown are representative arrangements of the 27 identified *gbsR* genes and their adjacent genes functionally associated with the synthesis of the compatible solute glycine betaine. A GbsB-like choline dehydrogenase (blue) (Boch et al., 1996) is encoded in the gene clusters of all members belonging to the genus *Bacillus*. Glycine betaine synthesis gene clusters of members of the genus *Staphylococcus* (Rosenstein et al., 1999) harbor a choline dehydrogenase, which is related to the BetA enzyme from *Escherichia coli* (gold) (Lamark et al., 1991). Genes encoding transport systems are depicted and color-coded according to the superfamily they belong to. *gbsU*-type genes (yellow) encode proteins with similarity to substrate binding proteins from ABC transporters (Scheepers et al., 2016), *cudT*-like genes (pink) encode a transporter of the betaine–choline–carnitine-transporter (BCCT) family (Ziegler et al., 2010), and genes for transporters of the sodium-solute-symporter (SSS) family (Bracher et al., 2016) are marked in green. In *H. halophilus* DSM 2266 (Burkhardt et al., 2009), the choline dehydrogenase gene has been labeled according to the nomenclature of the *gbsAB* gene cluster of *B. subtilis* (Boch et al., 1996); however, this gene actually encodes a BetA-type enzyme (Lamark et al., 1991). **(B)** Genetic organization of *opuA* gene clusters and sub-unit composition of the encoded OpuA-type ABC transporters. A total number of 18 genes for GbsR-like regulatory proteins (OpuAR) were identified, which are encoded in the immediate vicinity of gene clusters encoding an OpuA-type transporter (Kempf and Bremer, 1995; Obis et al., 1999; Wolters et al., 2010). Representative genetic arrangements of *opuA* gene loci are shown, and the sub-unit composition of the encoded OpuA-type ABC transporters is depicted. In three *opuA*-type gene clusters (e.g., in *P. larvae*), a single gene encodes the permease (OpuAB) and the substrate-binding domain (OpuAC) of the transporter. All of these fused transporters (van der Heide and Poolman, 2002) were present in members of the genus *Paenibacillus*.

Among the *gbsR*-containing microorganisms, both the *E. coli*- and *B. subtilis*-type of glycine betaine synthesis systems can be found (Figure 4A). In all members of the genus *Bacillus*, we find combinations of the gene for the type-III alcohol dehydrogenase GbsB with that encoding the GbsA glycine betaine aldehyde dehydrogenase; these genes are organized in an operon-type arrangement. The organization of this operon varies slightly, and examples for these gene clusters from *B. subtilis*, *Bacillus clausii*, *Bacillus licheniformis*, and *Bacillus megaterium* are depicted in Figure 4A. Interestingly, the corresponding gene clusters of *B. licheniformis* and *B. megaterium* contain a gene that encodes a membrane protein belonging to the sodium-solute-symporter family (SSS) (Bracher et al., 2016), and one can therefore speculate that these SSS-type transporters might mediate the import of choline into the cell for further oxidation to glycine betaine.

In the remaining microorganisms, represented in Figure 4A by *H. halophilus*, *Virgibacillus sp.* SK37, and *Staphylococcus xylosus*, *E. coli*-type genes (Lamark et al., 1991) for the synthesis of glycine betaine are found. Interestingly, a gene encoding a substrate-binding protein (GbsU) typically operating in conjunction with ABC transporters is present next to the glycine betaine synthesis genes in *H. halophilus* and *Virgibacillus* sp. SK37 (Figure 4A). However, genes encoding the other typical components of ABC transporters (Davidson et al., 2008) are absent from the immediate vicinity of the *gbsR–gbsA–gbsB* gene clusters present in these salt-tolerant bacteria. The amino acid-sequence-related GbsU lipoproteins from *H. halophilus* and *Virgibacillus* sp. *SK37* bear the hallmarks of compatible solute binding proteins because they carry in their predicted substrate-binding site (Supplementary Figure S2) an aromatic cage that allows the coordination of fully methylated head-groups of osmostress protectants via cation–π interactions (Schiefner et al., 2004a,b; Horn et al., 2006; Oswald et al., 2008; Smits et al., 2008; Wolters et al., 2010; Du et al., 2011; Pittelkow et al., 2011; Lang et al., 2015). Our *in silico* modeling of the *H. halophilus* and *Virgibacillus* sp. SK37 GbsU-binding proteins *via* the SWISS-MODEL web server (Waterhouse et al., 2018) identified the ProX protein from *Borrelia burgdorferi* (PDB accession-code: 3TMG) (a putative glycine betaine-binding protein) as the structural closet homolog of the GbsU substrate-binding proteins and revealed a ligand-binding site resembling in their architecture that of the *B. subtilis* OpuAC glycine betaine/proline betaine binding protein (Kempf and Bremer, 1995; Horn et al., 2006; Smits et al., 2008; Supplementary Figure S2).

Functional studies of the glycine betaine synthesis gene cluster from *H. halophilus* (Figure 4A) have already been conducted by Burkhardt et al. (2009) who found that the divergently oriented *gbsA-gbsB* and *gbsR-gbsU* genes are transcribed as choline-responsive operons (Burkhardt et al., 2009). The *H. halophilus* GbsR protein exhibits a 52% amino acid sequence identity to its *B. subtilis* GbsR counterpart. Functional studies with similar genetically configured glycine betaine synthesis genes (*gbsAB*) from *Halobacillus dabanensis* have also been performed, and *gbsR-gbsU*-type genes have also been found in this halophile, although a different nomenclature (*gbsI-gbsT*) has been used to annotate them (Gu et al., 2008).

Physiological studies and gene disruption analysis have also been conducted with the glycine betaine synthesis genes (*cudA-cudB*) from *S. xylosus* (Rosenstein et al., 1999; Figure 4A). The corresponding *gbsR*-type gene was named *cudC* and its encoded protein is 52% identical to the GbsR protein from *B. subtilis*. Next to the *cudC* gene, a choline transporter gene (*cudT*) is present (Rosenstein et al., 1999) that encodes a member of the BCCT-type transporter family; many of its members, including the choline transporter BetT from *E. coli* (Lamark et al., 1991) serve for the uptake of osmostress protectants (Ziegler et al., 2010). Notably, transcription of the *S. xylosus cudAB* glycine betaine biosynthetic genes is inducible by both high salinity and choline (Rosenstein et al., 1999). *S. xylosus* is generally regarded as non-pathogenic; choline import and glycine betaine synthesis gene clusters resembling those of *S. xylosus* (Figure 4A) can also be found in the pathogenic representatives (e.g., *Staphylococcus aureus*) of this genus (Figure 2).

In comparison with the *B. subtilis* GbsR protein (Nau-Wagner et al., 2012), GbsR-type proteins (27 representatives) whose genes are found in the immediate vicinity of genes for glycine betaine synthesis revealed an overall amino acid sequence identity ranging between 97 (for the GbsR protein from *Bacillus* sp. JS) and 41% (for the GbsR protein from *B. clausii* KSM-K16) (Supplementary Figure S3).

### *In silico* Assessment of GbsR-Type Regulatory Genes Associated With Genes for Osmostress Protectant Uptake Systems

Our bioinformatics approach identified 38 homologs of GbsR, which are associated with transport systems most likely involved in osmostress protectant uptake. These genes are all associated with genes encoding ABC transporters related to the well-studied OpuA, OpuB, and OpuC systems of *B. subtilis* (Hoffmann and Bremer, 2016, 2017). *B. subtilis* has three copies of *gbsR*-type genes; one of them (*gbsR*) is associated with the *gbsAB* operon, and the two other copies are associated with the *opuB* and *opuC* gene clusters that encode ABC transporters for the import of the glycine betaine precursor choline and other types of osmostress protectants (Kappes et al., 1999; Nau-Wagner et al., 2012; Lee et al., 2013; Hoffmann and Bremer, 2017; Teichmann et al., 2017). No *gbsR*-type gene is associated in *B. subtilis* with the genes encoding the OpuA transporter (Kempf and Bremer, 1995; Nau-Wagner et al., 2012).

We found in our database search *gbsR*-type regulators (20 out of 179) associated with genes for OpuB- and OpuC-type transporters, and these are phylogenomically narrowly restricted to members of the genus *Bacillus* (Figure 2). While this type of genetic association was expected from previous studies (Kappes et al., 1999; Nau-Wagner et al., 2012; Lee et al., 2013), we detected a substantial group (18 out of 179) of *gbsR*-type genes in the immediate vicinity of genes encoding OpuA-type ABC transporters (Kempf and Bremer, 1995; Hoffmann and Bremer, 2017; Figure 2). The *B. subtilis* OpuA transporter consists of an ATPase (OpuAA), the trans-membrane component OpuAB, and the substrate-binding protein OpuAC (Kempf and Bremer, 1995; Horn et al., 2006; Smits et al., 2008), a lipoprotein tethered to the outer face of the cytoplasmic membrane (Kempf et al., 1997). Variants of the OpuA system exist in which the substrate-binding protein is fused to the *trans*-membrane domain of the ABC transporter (van der Heide and Poolman, 2002); e.g., the OpuA system from *Lactococcus lactis* (Obis et al., 1999; Mahmood et al., 2006; Wolters et al., 2010) and various representatives of the *Bacillus* genus (Teichmann et al., 2018). We found both types of OpuA transporters in our dataset, and these are primarily present in *Paenibacillius* (Figure 2). The genetic organization of the *opuA*-type operons and the predicted sub-unit composition of the encoded ABC transporters are depicted in Figure 4B for representative examples (e.g., from *B. infantis*, *P. larvae*). These two microorganisms belong to the Bacillales but inhabit different ecological niches. *B. infantis* is a marine bacterium isolated from the Gulf of Mexico (Siefert et al., 2000), but strains of this genus can rarely also be found in clinical material isolated from humans (Massilamany et al., 2016). *P. larvae* is an entomopathogen and the etiological agent of the American Foulbrood, a deadly disease of honey bees (Dingman, 2017). We conducted *in silico* modeling studies with the substrate-binding proteins from these two microorganisms and found that both of them possesses a predicted ligand-binding site resembling the aromatic cage of the *B. subtilis* and *L. lactis* OpuAC glycine betaine and proline betaine substrate-binding proteins (Horn et al., 2006; Smits et al., 2008; Wolters et al., 2010; Supplementary Figure S4).

To study the relationship of the proteins encoded by the various groups of *gbsR*-type genes, we aligned their amino acid sequences using MAFFT (Katoh et al., 2017) and conducted a clade analysis using the bioinformatics resources provided via the iTOL web-server (Letunic and Bork, 2016). The corresponding data summarized for the 179 GbsR-type proteins in Supplementary Figure S5 demonstrate that the genetic affiliation of *gbsR*-type genes with particular gene neighborhoods is reflected in their position in the GbsR protein-based tree. When the amino acid sequences of those 65 GbsR-type proteins that are associated with putative glycine betaine biosynthetic genes and those for OpuA-, OpuB-, and OpuC-type compatible solute ABC transporters were aligned, those residues forming the aromatic cage-like structure implicated in inducer binding (Nau-Wagner et al., 2012) are notably well conserved (Supplementary Figure S3). Among these GbsR-type proteins, six residues that are part of the winged helix-turn-helix DNA-binding motif (Figures 1B,C) are also well conserved, and three strictly conserved residues are found in the linker region (Supplementary Figure S3).

Relative to the *B. subtilis* GbsR protein (Nau-Wagner et al., 2012), the overall amino acid sequence identity of GbsR-type proteins associated with OpuA-type transporters ranges between 41 and 28%, those for GbsR-type proteins associated with OpuB-type transporters ranges between 35 and 34%, and those for GbsR-type proteins associated with OpuC-type transporters ranges between 34 and 29% (Supplementary Figure S3).

In contrast, an alignment of the amino acid sequences of those 80 GbsR-type proteins that are not functionally associated with cytochrome *bd*-type oxygen reductase gene clusters or cellular osmostress response systems showed that these regulatory proteins are far less well conserved (Supplementary Figure S6). In this heterogeneous group of GbsR-type proteins, the corresponding genes are either associated with transport systems with various predicted substrate specificities or genes whose physiological function cannot readily predicted (Figure 2). Notably, the vast majority (27/29) of the 29 archaeal GbsR-type proteins fall into this latter class (Figure 2). These 80 GbsR-type proteins possess an overall amino acid sequence identity in comparison with the *B. subtilis* GbsR protein that ranges between 51 and 21% (Supplementary Figure S6).

### Functional Characterization of the OpuA Osmostress Protectant Uptake System From *B. infantis*

**FIGURE 5:**
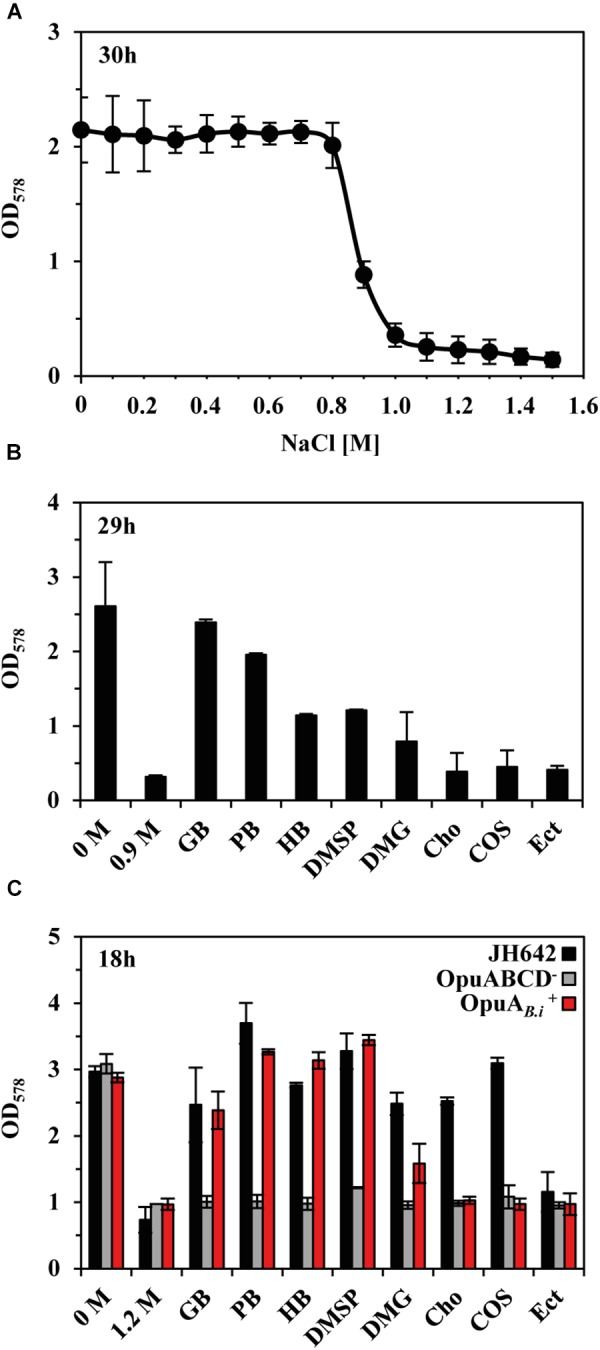
Protection of *B. infantis* NRRL B-14911 against osmotic stress. **(A)** Influence of increasing salinity on the growth of *B. infantis* NRRL B-14911. Cultures were grown in basal medium adjusted to the indicated salinity. Growth yields were measured after 30 h of incubation at 37°C. **(B)** Osmostress protection assays were carried out in basal medium supplemented with 0.9 M NaCl and in the presence of 1 mM of the indicated osmostress protectants. After 29 h of incubation at 37°C, growth yields were determined by measuring the OD_578_ of the cultures. **(C)** Substrate specificity of the *B. infantis* OpuA transporter. The *opuA* operon (and its flanking *opuAR* gene) of *B. infantis* NRRL B-14911 was heterologously expressed in a derivative of *B. subtilis* JH642, lacking all uptake system for compatible solutes except for the L-proline-specific OpuE transporter (Hoffmann and Bremer, 2017; Teichmann et al., 2017). Growth assays were conducted in minimal medium (SMM), either in the absence or presence of 1.2 M NaCl and 1 mM of the indicated osmostress protectants. After 18 h of incubation at 37°C, the optical densities (OD_578_) of the cultures were measured. The given values are the means and standard deviations of four independent biological replicates.

Uptake and synthesis of compatible solutes have been intensively studied in *B. subtilis* (Hoffmann and Bremer, 2016, 2017), a soil isolate (Earl et al., 2008). However, relatively little is known with respect to this ecophysiologically important topic in *Bacilli* that inhabit marine ecosystems (Siefert et al., 2000). To experimentally support our bioinformatics analysis of GbsR-type regulators associated with genes encoding transporters for osmostress protectants, we focused our further analysis on the OpuA system of the marine isolate *B. infantis* NRRL B-14911 (Siefert et al., 2000; Massilamany et al., 2016). As an initial step, we assessed the growth properties of *B. infantis* NRRL B-14911 in a chemically defined basal medium with various salinities. This strain grew well up to a NaCl concentration of 0.8 M (Figure 5A). Sea-water typically contains about 0.6 M NaCl and the observed level of salt tolerance of *B. infantis* NRRL B-14911 is thus in accord with the marine origin of this isolate (Siefert et al., 2000).

Next, we studied the possible osmostress protection of *B. infantis* NRRL B-14911 by various exogenously provided compatible solutes. For this experiment, we grew cultures of *B. infantis* NRRL B-14911 in basal medium with 0.9 M NaCl in the absence or presence of 1 mM of such osmostress protectants. Growth medium containing 0.9 M NaCl strongly inhibits the proliferation of *B. infantis* NRRL B-14911 (Figures 5A,B), and the presence of either glycine betaine or proline betaine afforded a substantial level of osmostress protection (Figure 5B). A moderate level of osmostress protection was achieved by adding the nitrogen-containing compatible solutes homobetaine and dimethlyglycine (DMG) and the sulfur-containing osmolyte dimethylsulfoniopropionate (DMSP), a compatible solute found widely in marine ecosystems (Broy et al., 2015), to the growth medium (Figure 5B). Since the genome sequence of *B. infantis* NRRL B-14911 (Massilamany et al., 2016) lacks glycine betaine synthesis genes, it is readily understandable why choline is not osmostress protective (Figure 5B), as this compound is not a compatible solute *per se* because its osmostress-relieving properties depend on its enzymatic conversion into glycine betaine (Boch et al., 1996).

The genome sequence of *B. infantis* NRRL B-14911 predicts in addition to OpuA, the presence of several other types of osmostress protectant uptake systems (e.g., OpuF, OpuD, OpuE) (Teichmann et al., 2018), a feature that precludes an assignment of a defined substrate spectrum to the OpuA ABC transporter. We therefore cloned the *opuA* gene cluster (*opuAR/opuAA-opuAB-opuAC*) (Figure 4B) and inserted it as a single copy into the chromosomal *amyE* gene of a *B. subtilis* chassis strain (Teichmann et al., 2017) with defective OpuA, OpuB, OpuC, and OpuD systems (this strain possesses the L-proline transporter OpuE). The resulting recombinant *B. subtilis* strain CAB2 (Supplementary Table S1) was protected from the detrimental effects of high salinity (1.2 M NaCl) by the added compatible solutes glycine betaine, proline betaine, homobetaine, DMSP, and at a reduced level, also by DMG (Figure 5C). Hence, this substrate profile of the *B. infantis* NRRL B-14911 OpuA transporter is similar to that of the corresponding system from *B. subtilis* (Hoffmann and Bremer, 2016, 2017).

### Transcriptional Regulation of the *B. infantis opuA* Gene Cluster via the GbsR-Type Regulator OpuAR

Having established that the *opuA* gene cluster from *B. infantis* NRRL B-14911 was functionally expressed in the heterologous *B. subtilis* host strain we focused on the role of the *B. infantis* GbsR protein in this process. To distinguish its annotation from the three GbsR-type regulators [GbsR, OpcR (YvbF), YvaV] found in *B. subtilis* (Nau-Wagner et al., 2012; Lee et al., 2013) and the CydE GbsR-type regulator (Xia et al., 2018), we refer in the following to the *B. infantis* GbsR protein as the OpuAR regulatory protein.

**FIGURE 6:**
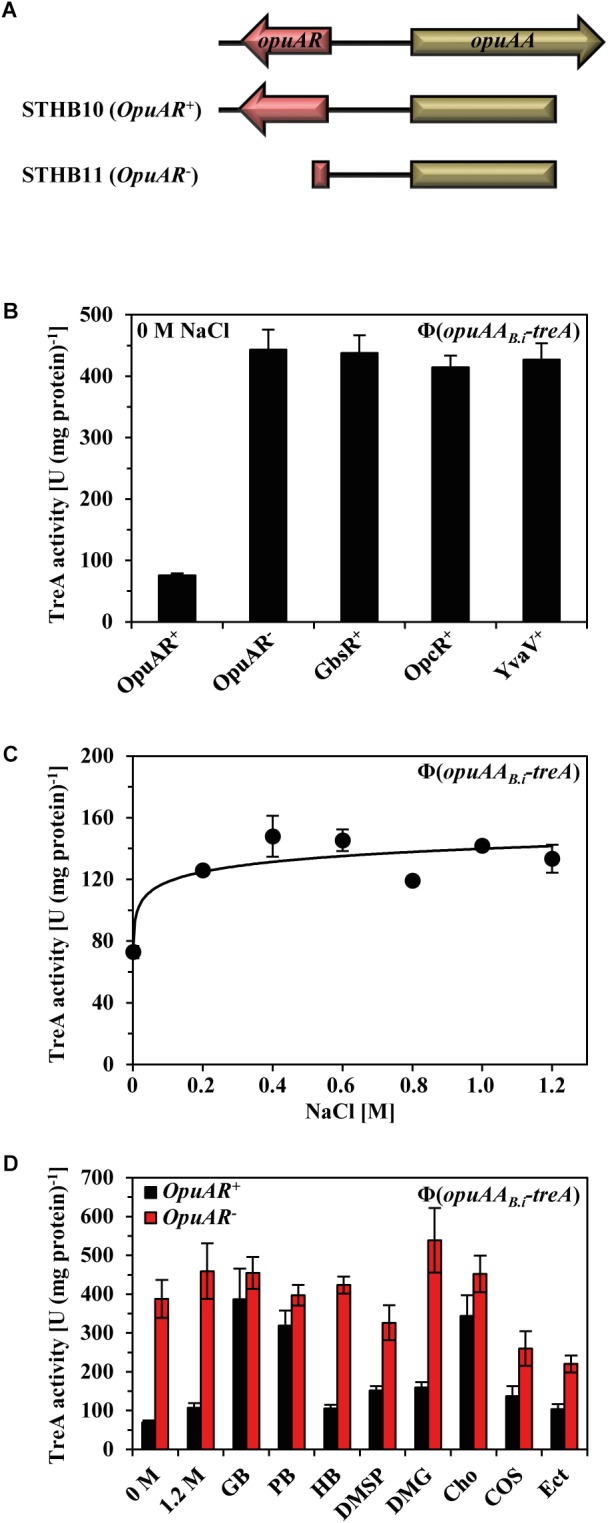
Influence of the *B. subtilis* GbsR-like regulators on the expression of *opuA* from *B. infantis* and its transcriptional response to the presence of compatible solutes. **(A)** Genetic structure of the *opuAA-treA* transcriptional reporter fusions. **(B)** The *opuAA-treA* reporter gene fusions, either including or lacking the *B. infantis opuAR* gene, were integrated into the chromosome of the *B. subtilis* strain STHB05; it carries gene disruption mutations of all three GbsR-like regulators, resulting in strains STHB10 (OpuAR^+^) and STHB11 (OpuAR^-^). A *opuAA-treA* reporter gene fusions lacking the *B. infantis opuAR* gene was introduced into the chromosome of strains possessing only one of the GbsR-type genes present in *B. subtilis*: strain STHB67 (GbsR^+^), strain STHB65 (OpcR^+^), strain STHB66 (YvaV^+^). The *opuAA-treA* reporter strains were grown in SMM to early exponential growth phase (OD_578_ 1–1.5) and then assayed for TreA reporter enzyme activity. The shown data represent four independent biological replicates and each culture was assayed twice. **(C)** Strain STHB10 (*opuAR*^+^/*opuAA-treA*) was grown in SMM with increasing NaCl concentrations until each of these cultures reached mid-exponential growth phase (OD_578_ of about 1.5) and were then harvested for TreA reporter enzyme activity assays. The given data are the means and standard deviations of four independent biological replicates and each culture was assayed twice. **(D)** Expression of the *opuAA-treA* reporter gene construct in response to extracellular provided compatible solutes. The *opuAA-treA* reporter fusion strains STHB10 (OpuAR^+^) and STHB11 (OpuAR^-^) were cultivated in SMM containing 1.2 M NaCl either in the absence or presence of 1 mM of the indicated compatible solutes. The *opuAA-treA* reporter fusion strains were grown to early exponential growth phase (OD_578_ 1–1.5) and then assayed for TreA reporter enzyme activity. The given data are the means and standard deviations of four independent biological replicates and each culture was assayed twice.

To study the transcriptional regulation of the *B. infantis* NRRL B-14911 *opuA* gene cluster, we constructed two transcriptional reporter fusions to the gene (*treA*) for the salt-tolerant enzyme phospho-α-(1,1)-glucosidase (TreA) whose enzyme activity can readily be photometrically assayed with the chromogenic substrate α-PNPG (Gotsche and Dahl, 1995). In one of these *opuAA-treA* reporter fusion constructs, the up-stream located *opuAR* gene was present, while it was incomplete in the other reporter fusion construct (Figure 6A). These two reporter fusions were then inserted as a single copy into the *amyE* gene in the chromosome of a *B. subtilis* strain lacking all native GbsR-type proteins. Disruption of the *opuAR* gene resulted in a strong de-repression in the transcription of the *opuAA-treA* reporter fusion (Figure 6B) thus demonstrating that OpuAR genetically serves as a repressor. The *B. subtilis* GbsR, OpcR, and YvaV regulatory proteins could not functionally substitute the repressor activity of the *B. infantis* OpuAR protein when their influences were separately tested with the *opuAA-treA* fusion construct lacking an intact *opuAR* gene (Figure 6B).

Transcription of genes encoding uptake systems for compatible solutes is typically induced in response to increase in the external salinity (Lucht and Bremer, 1994; Wood, 1999, 2011; Bremer and Krämer, 2000; Wood et al., 2001; Krämer, 2010; Hoffmann and Bremer, 2016, 2017). However, a sustained increase in the salinity of the growth medium afforded only very moderate increases in the expression of an *opuAA-treA* reporter fusion strain carrying at the same time the *B. infantis opuAR* gene (Figure 6C).

Choline serves as the inducer for relief of GbsR-mediated repression of the *B. subtilis gbsAB* and *opuB* operons, and binding of choline to the repressor protein (*K*_d_ = 165 ± 15 μM) has been measured with affinity-purified GbsR via changes in the intrinsic Trp-fluorescence upon ligand binding (Nau-Wagner et al., 2012). To identify possible effector molecules for the *B. infantis* OpuAR protein, we conducted a series of *in vivo* experiments in which we assessed any possible inducing effects of compatible solutes on *opuAA-treA* transcriptional activity in an OpuAR-dependent fashion (Figure 6D). In a *B. subtilis* strain possessing all native Opu transport systems, glycine betaine, proline betaine, and choline served as inducers of *opuAA-treA* reporter activity, while all other tested compatible solutes (DMG, DMSP, homobetaine, choline-*O*-sulfate, and ectoine) did not serve as inducers (Figure 6D). Hence, by comparing the data from the osmostress protection assays (Figure 5C) with those of the *opuAA-treA* reporter study (Figure 6D), it becomes apparent that major substrates (glycine betaine and proline betaine) of the *B. infantis* OpuA ABC importer also serve as inducers of OpuAR to relieve the DNA-binding activity of this repressor protein. However, this dataset also holds several surprises: (i) DMSP, which is a good substrate for the *B. infantis* OpuA transporter (Figure 5C) is not an inducer of OpuAR (Figure 6D). (ii) Conversely, choline, which is not imported via the *B. infantis* OpuA system (Figure 5C), acts as a strong inducer of OpuAR (Figure 6D).

**FIGURE 7:**
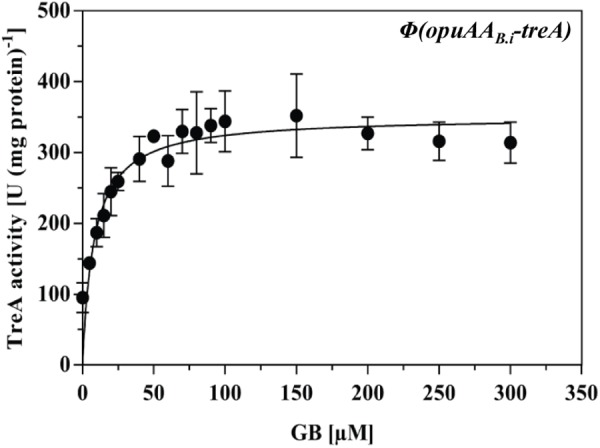
Expression of *opuA* from *B. infantis* in response to various glycine betaine concentrations in a *B. subtilis* chassis strain. The *opuAA-treA* reporter strain STHB10 (*opuAR*^+^/*opuAA-treA*) was grown in SMM containing 1.2 M NaCl and various concentrations of glycine betaine ranging between 0 and 300 μM until each of these cultures reached mid-exponential growth phase (OD_578_ of about 1.5) and were then harvested for TreA reporter enzyme activity assays. The given data are the means and standard deviations of two independent biological replicates and each culture was assayed twice.

Compatible solutes are typically present in natural habitats of microorganisms in rather low concentrations (Bouskill et al., 2016). They are released into the environment by osmotically down-shocked or ruptured microbial cells (Hoffmann et al., 2008, 2012), through root exudates and rotting plant material in terrestrial systems and by algae in marine habitats (Welsh, 2000). To study the sensitivity of the transcriptional response of the *B. infantis opuA* gene cluster to the presence of an inducer, we performed a dose–response experiment in which we provided a range of glycine betaine concentrations (from 5 to 300 μM) to high osmolarity-grown cells of a *B. subtilis* reporter strain carrying a chromosomal *opuAR*^+^-*opuAA-treA* reporter construct. The addition of as little as 5 μM glycine betaine to the growth medium already triggered a noticeable increase in transcriptional activity and a concentration of just 75 μM glycine betaine was sufficient to fully induce the expression of the reporter fusion (Figure 7).

### Quaternary Assembly of the OpuAR Protein and Mutational Analysis of Its Putative Inducer-Binding Site

**FIGURE 8:**
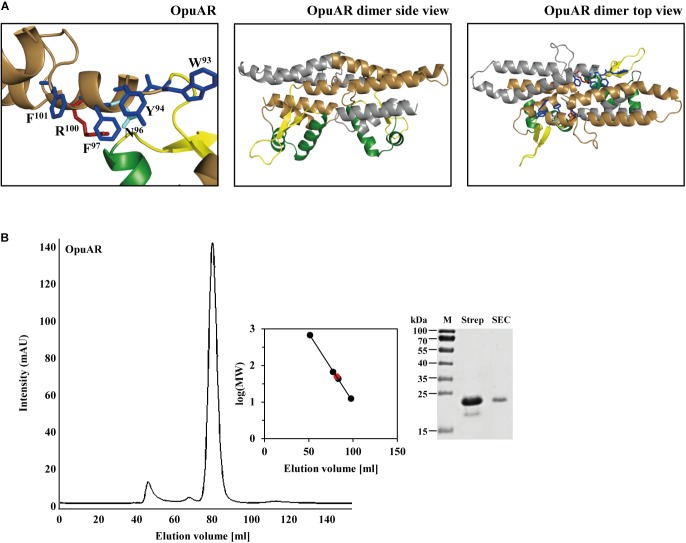
*In silico* model of the *B. infantis* OpuAR protein and analysis of its quaternary assembly. **(A)** *In silico* models of the monomer and dimers of the *B. infantis* OpuAR protein; they are based on the crystal structure of the DNA-binding protein Mj223 of *M. jannaschii* (PDB entry 1KU9) (Ray et al., 2003). The winged helix-turn-helix region is represented in green, the inter-domain linker region is shown in yellow, and the four aromatic amino acids putatively involved in structuring the inducer-binding site are highlighted in blue. The two amino acids (N^96^ and R^100^) in OpuAR deviating from the amino acid composition of the aromatic cage found in the *B. subtilis* GbsR protein (Nau-Wagner et al., 2012) are highlighted. **(B)** Size-exclusion chromatography of the affinity-purified *B. infantis* OpuAR protein and analysis of OpuAR by SDS–PAGE. Immediately after the purification of the OpuAR-Strep-tag II recombinant protein, a 2-ml protein solution (1.5 mg ml^-1^) was loaded onto a size-exclusion chromatography column (HiLoad 16/600 Superdex 200 pg) that was run in a buffer containing 100 mM KPP (pH 8) and 300 mM NaCl. Thyroglobulin (667 kDa), albumin (66 kDa), ovalbumin (43 kDa), and cytochrome C (12.4 kDa) were used to standardize the size-exclusion chromatography column. The purity and molecular mass of the OpuAR protein subsequent to size-exclusion chromatography was assessed by SDS-polyacrylamide gel electrophoresis (15%); proteins were stained with Coomassie Brilliant Blue. 5 μg of the affinity purified OpuAR protein (Strep), and 1 μg of the OpuAR protein passed through the size-exclusion chromatography (SEC) column was loaded onto the SDS-polyacrylamide gel.

The *M. jannaschii* Mj223 protein (Ray et al., 2003) from which the *B. subtilis* GbsR (Figures 1A–C) and the *B. infantis* OpuAR (Figure 8A) *in silico* models are derived is a homo-dimer in the crystal structure (PDB accession code: 1KU9; Ray et al., 2003). To assess if the *B. infantis* OpuAR protein adheres to the common dimeric-fold of MarR-type regulators (Deochand and Grove, 2017; Grove, 2017), we performed a size-exclusion experiment with affinity-purified OpuAR. The data documented in Figure 8B show that the *B. infantis* OpuAR protein forms a stable dimer in solution.

**FIGURE 9:**
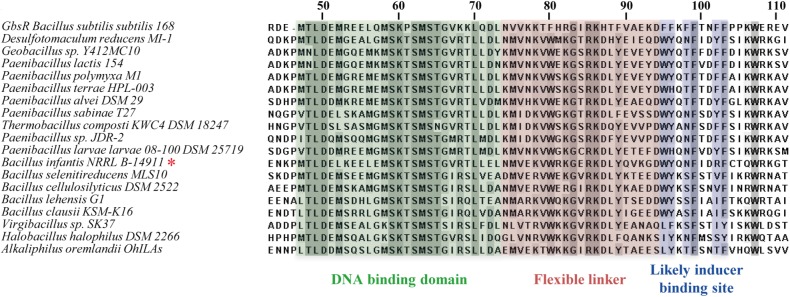
Amino acid sequence alignment of OpuAR-type proteins. The amino acid sequences of 18 OpuAR-type proteins were aligned with the aid of the MAFFT server (Katoh et al., 2017) and compared to the amino acid sequence of the *B. subtilis* GbsR protein (Nau-Wagner et al., 2012). Only the N-terminal domain of these proteins is depicted. Highly conserved amino acids are shaded in gray. The segments of the GbsR-/OpuAR-type proteins corresponding to the winged helix-turn-helix motif (green), the inter-domain linker (reddish), and of the putative inducer-binding site (blue) are highlighted. A red star (_∗_) marks the position of the *B. infantis* NRRL B-14911 OpuAR protein in the amino acid sequence alignment.

In Figure 9, we have compiled and aligned the amino acid sequence of all OpuAR-type proteins that we identified in the course of the bioinformatics analysis of microbial genome sequences (Figure 2). These proteins have an overall degree of amino acid sequence identity ranging between 51 (*D. reducens* MI-1) and 37% (*A. oremlandii* OhlLAs) when the *B. infantis* OpuAR protein was used as a benchmark. In this alignment (only the N-terminal domain is shown in Figure 9), we have highlighted the winged helix-turn-helix region of the GbsR/OpuAR DNA-binding domain, the flexible linker connecting the DNA reading head with the dimerization domain and the putative inducer-binding site (Figures 1B,C). In these regions, some amino acids are either strictly or highly conserved (Figure 9), and this is in particularly notable in the putative inducer-binding site (Nau-Wagner et al., 2012). In inspecting the amino acids forming the putative inducer-binding site in the GbsR protein (Figures 1A,C), six aromatic residues are present in this domain of GbsR from *B. subtilis*, while only four are found in the OpuAR protein from *B. infantis* NRRL B-14911 (Figures 8A, 9). These non-conserved positions correspond to Asn^96^ and Arg^100^ in the *B. infantis* OpuAR protein.

Fluorescence spectroscopic ligand-binding assays conducted previously demonstrated the binding of choline by the purified *B. subtilis* GbsR protein with a *K*_d_ value of 165 ± 15 μM, but GbsR does not bind glycine betaine (Nau-Wagner et al., 2012). To compare the ligand binding characteristics of the *B. infantis* OpuAR protein with that of the *B. subtilis* GbsR protein, we purified a recombinant version of the OpuAR protein carrying a *Strep*-tag II affinity peptide at its carboxy-terminus (Figure 8B). We then used this purified protein for ligand-binding assays by employing fluorescence spectroscopy and determined a *K*_d_ of 193 ± 40 μM for choline (Table 1), a value similar to that previously reported for the *B. subtilis* GbsR protein (Nau-Wagner et al., 2012).

**Table 1:** Binding of choline and glycine by purified OpuAR protein and its variants.

Amino acid in OpuAR	93	94	96	97	100	101	*K*_d_ (μM)^a^	
							Choline	Glycine betaine
GbsR	F	F	F	F	F	F	165 ± 15^b^	–
OpuAR	W	Y	N	F	R	F	193 ± 40	301 ± 24
W^93^F	F	Y	N	F	R	F	210 ± 33	224 ± 24
Y^94^F	W	F	N	F	R	F	240 ± 33	293 ± 37
N^96^F	W	Y	F	F	R	F	262 ± 34	254 ± 39
R^100^F	W	Y	N	F	F	F	100 ± 15	303 ± 40
Y^94^F/R^100^F	W	F	N	F	F	F	61 ± 11	70 ± 12

*^a^Changes in the intrinsic tryptophan fluorescence of the purified B. infantis NRRL B-14911 OpuAR protein and its mutant derivatives were used to determine the affinity (K_d_) of OpuAR to the ligands choline and glycine betaine using a procedure previously applied to quantitate ligand binding by the B. subtilis GbsR protein (Nau-Wagner et al., 2012), or substrate-binding proteins from ABC transporters for compatible solutes (Horn et al., 2006; Oswald et al., 2008; Smits et al., 2008; Pittelkow et al., 2011). The data shown are derived from one OpuAR protein batch, with each of the substrate concentrations used for the K_d_-measurement (choline: 25–1,600 μM; glycine betaine: 25–1,300 μM) assayed in triplicate. ^b^The K_d_-value given for choline binding by the B. subtilis GbsR protein was taken from the literature; this protein does not bind glycine betaine (Nau-Wagner et al., 2012).*

To assess the differences in the amino acid sequence composition of the putative inducer binding sites in GbsR and OpuAR (Figures 1A, 8A), we constructed via site-directed mutagenesis variants of the OpuAR protein in which we either conservatively substituted Trp^93^ or Tyr^94^ by a Phe residue. These single amino acid substitutions had marginal effects on the choline-binding activity of OpuAR (Table 1). Similarly, no strong effect on choline binding was observed for an OpuAR variant in which we changed Asn^96^ to a Phe residue (Table 1). However, a notable improvement in affinity for the inducer choline was observed in an *opuAR* mutant in whom we replace the positively charged Arg^100^ with a Phe residue (Table 1). By combining the Tyr^94^ to Phe and the Arg^100^ to Phe mutations, and thus creating an aromatic cage resembling that of the *B. subtilis* GbsR protein (Figure 1A), choline-binding activity improved notably by about threefold (Table 1).

In contrast to GbsR (Nau-Wagner et al., 2012), the *B. infantis* OpuAR protein was able to bind glycine betaine (*K*_d_ value of 301 ± 24 μM), albeit with a somewhat reduced affinity in comparison with choline (Table 1). The single amino acid substitutions in OpuAR described above had in essence either no or only marginal effects on glycine betaine binding, except in the Tyr^94^ to Phe and the Arg^100^ to Phe double mutant in which the *K*_d_ value was fourfold improved to 70 ± 12 μM (Table 1).

## Discussion

Two GbsR-type proteins (GbsR, OpcR) have previously been functionally associated with osmostress adjustment response systems of *B. subtilis*. These control the transcription of genes for the synthesis of the compatible solute glycine betaine from the precursor choline and of transporters for the import of various types of osmostress protectants (Nau-Wagner et al., 2012; Lee et al., 2013; Hoffmann and Bremer, 2016). The detailed phylogenomic analysis of GbsR-type proteins reported here (Figure 2) now shows that the corresponding structural genes are indeed frequently associated in different microbial taxa with various types of glycine betaine synthesis gene clusters and with genes that could encode OpuA-, OpuB-, and OpuC-type ABC importers for osmostress protectants (Figures 2, 4A).

However, *gbsR*-type genes are also found in other genetic contexts. A substantial sub-group (34 out of 179) of *gbsR* genes is present in the immediate vicinity of *cydAB*-type or *cydABCD*-type gene clusters (Figure 3) encoding oxygen reductases (Borisov et al., 2011; Degli Esposti et al., 2015). Very recently, and consistent with our independently performed phylogenomic analysis (Figures 2, 3), Xia et al. (2018) reported that the expression of the *cydAB* operon from *Alishewanella* sp. WH16-1 is negatively regulated by a GbsR-type regulator, to which the authors referred to as CydE (Xia et al., 2018). GbsR-type proteins are also found in several genera of *Archaea* (Figure 2). In this sub-group, and that formed by the Bacteroidetes (Figure 2), no consistent picture emerges from the gene neighborhood analysis with respect to the physiological process that might be regulated by GbsR-type proteins. Importantly, among the archaeal representatives of this heterogeneous group, we find the gene encoding the *M. jannaschii* Mj223 protein whose crystal structure (Ray et al., 2003) can probably serve as a general template for predicting (Nau-Wagner et al., 2012) the overall tertiary structure of homodimeric GbsR-type regulators (Figures 1A–C, 8A). Consistent with the *in silico* model of the *B. infantis* OpuAR protein (Figure 8A), our assessment of the OpuAR quaternary structure by size exclusion chromatography showed that this regulatory protein is indeed a dimer in solution (Figure 8B). The *M. jannaschii* Mj223 crystal structure (Ray et al., 2003) and the derived GbsR (Nau-Wagner et al., 2012) and OpuAR (this study) *in silico* models classify GbsR-type proteins as members of the MarR superfamily of regulators. This group of transcription factors contains both activators and repressors (Deochand and Grove, 2017; Grove, 2017). The so-far four genetically and functionally studied members of the GbsR family (GbsR: Nau-Wagner et al., 2012; OpcR: Lee et al., 2013; CydE: Xia et al., 2018; and OpuAR: this study; Figures 6B,D) all serve as repressors.

We found in our database analysis many examples of *gbsR*-type genes that are present in the immediate vicinity of glycine betaine biosynthesis genes and genes for OpuB- and OpuC-type transporters (Figure 2), a genetic arrangement expected from previous studies with *B. subtilis* (Nau-Wagner et al., 2012; Lee et al., 2013). However, the presence of *gbsR*-type genes next to *opuA*-type gene clusters (Kempf and Bremer, 1995) was a novel finding. We therefore focused our work on this group of genes in order to assess whether there is not only a physical association between the *opuA* operon and *gbsR*-type genes (Figure 2) but whether this genetic arrangement also reflects a functional relationship. Since marine isolates of the genus *Bacillus* (Siefert et al., 2000) are not well studied with respect to their salt stress response, we chose *B. infantis* NRRL B-14911 (Siefert et al., 2000; Massilamany et al., 2016) as a model strain to address this question.

In contrast to the *B. subtilis opuA* operon (Kempf and Bremer, 1995; Hoffmann et al., 2013), transcription of the *B. infantis opuA* gene cluster is not strongly inducible by salt stress, at least not in the heterologous *B. subtilis* host (Figure 6C). The observation that salt stress has no major effects on the level of *opuA* transcription in *B. infantis* (Figure 6C) might be connected with the fact that this bacterium lives in a permanent high salinity marine ecosystem (Siefert et al., 2000). Instead, *opuA* transcription is placed under the negative control of the GbsR-type regulator OpuAR (Figures 6B,D) and it is induced *in vivo* by the compatible solutes glycine betaine, proline betaine, and choline (Figure 6D). The genetic disruption of the *opuAR* gene results in a strong de-repression of *opuA* transcription and the loss of the OpuAR repressor cannot be functionally replaced by any of the three GbsR-type proteins (GbsR, OpcR, YvaV) found in *B. subtilis* (Figure 6B). This is consistent with data reported for the *B. subtilis* GbsR and OpcR proteins with respect to their different effects on the transcription of the *gbsAB*, *opuB*, and *opuC* operons (Nau-Wagner et al., 2012; Lee et al., 2013). It thus appears that significant functional differences in either the DNA-recognition element, a winged helix-turn-helix (Ray et al., 2003; Nau-Wagner et al., 2012; Deochand and Grove, 2017; Grove, 2017), of GbsR-type regulators and/or in their respective operator sites must exist (Nau-Wagner et al., 2012; Lee et al., 2013; Xia et al., 2018).

A dose–response experiment revealed how exquisitely sensitive the OpuAR/*opuA* promoter regulatory system acts to trigger enhanced *opuA* gene expression once an inducer (e.g., glycine betaine) is present in the surroundings of the *Bacillus* cells (Figure 7). The induction of genes encoding import systems for osmostress protectants are, with the exception of those involved in the uptake of the precursor choline for glycine betaine synthesis (Lamark et al., 1991; Nau-Wagner et al., 2012; Chen et al., 2013; Wargo, 2013; Meadows and Wargo, 2018), not substrate-inducible. Instead, high osmolarity typically triggers their enhanced expression (Lucht and Bremer, 1994; Wood, 1999; Bremer and Krämer, 2000; Wood et al., 2001; Krämer, 2010; Hoffmann and Bremer, 2016, 2017). The weak transcriptional response of *opuA_B.i_* to high salinity (Figure 6C) and its strong induction by various compatible solutes (Figures 6D, 7) are thus quite unusual given that *B. infantis* cannot use glycine betaine as a nutrient (Supplementary Figure S7). To the best of our knowledge, we are not aware of any other example where the genes for a compatible solute uptake system functioning exclusively in cellular osmostress adjustment, and not for their exploitation as nutrients, are regulated in this way. However, in the pathogens *Pseudomonas aeruginosa* and *Pseudomonas syringae* complex regulatory circuits exists that control intertwined systems for the import and synthesis of selected osmostress protectants and the catabolism of these quaternary ammonium compounds, where for instance, choline and glycine betaine serve as inducers (Chen et al., 2013; Wargo, 2013; Meadows and Wargo, 2018).

The purified OpuAR protein binds both glycine betaine and choline (Table 1), solutes that also served *in vivo* as its inducers (Figures 6D, 7). Recognition of choline as an effector molecule for OpuAR is surprising because this compound is not a substrate for the OpuA_B_*_.i_* transporter and it also does not serve as an osmostress protectant for *B. infantis* NRRL B-14911 (Figures 5B,C), consistent with the fact that its genome sequence (Massilamany et al., 2016) lacks genes for glycine betaine synthesis from choline. The OpuAR protein differs in its inducer profile (Figure 6D) from that of the *B. subtilis* GbsR protein (Nau-Wagner et al., 2012) as it can not only bind choline but also glycine betaine with similar affinities (Table 1). Because choline cannot be exploited as an osmostress protectant, its function as an OpuAR inducer might simply reflect the architectural similarities of the ligand-binding sites present in choline and glycine betaine substrate-binding proteins (Schiefner et al., 2004a,b; Horn et al., 2006; Oswald et al., 2008; Du et al., 2011; Pittelkow et al., 2011). There is precedent for chemically closely related compounds to serve as gratuitous inducers. For instance, although the compatible solute proline betaine cannot be catabolized by *B. subtilis*, the proline-responsive PutR activator protein recognizes it as an effector molecule to induce expression of the *putBCP* proline import and catabolism genes (Moses et al., 2012).

There is an important distinction between GbsR-type proteins functionally associated with cellular defense against osmotic stress (Figure 2) and those representing the other two major sub-groups of this protein family (Supplementary Figure S5). A cluster of aromatic amino acids is consistently present in GbsR-type proteins that do belong to the group of osmostress-associated regulators (GbsR, OpuAR, OpcR, and YvaV) (Supplementary Figure S3), while it is absent in the other GbsR-type proteins (Supplementary Figure S6). This *in silico* analysis therefore suggests that the effector molecules for the osmostress-associated group of GbsR/OpuAR/OpcR/YvaV-type proteins are distinct from that of the other two major groups of the GbsR family. Indeed, the data reported by Xia et al. (2018) for the *Alishewanella* CydE repressor, which lacks the residues for the formation of an aromatic cage, demonstrate that this GbsR-type regulatory protein uses sulfate as its inducer (Xia et al., 2018).

Building on the architecture of compatible solute-binding proteins (Figures 1D–F) and the *in silico* models of GbsR and OpuAR (Figures 1A–C, 8A), an aromatic cage seems to be the prime candidate for inducer binding by GbsR-type proteins functionally associated with cellular osmostress defense systems. Aromatic cages of slightly different architectures have been found in many substrate binding proteins operating in conjunction with osmolyte ABC-type import systems present in *Bacteria* and *Archaea* (Schiefner et al., 2004a,b; Horn et al., 2006; Oswald et al., 2008; Smits et al., 2008; Wolters et al., 2010; Du et al., 2011; Pittelkow et al., 2011; Lang et al., 2015). A similarly configured ligand-binding site is also present in the single-component BCCT-type glycine betaine transporter BetP from *Corynebacterium glutamicum* (Ziegler et al., 2010; Perez et al., 2014). The architecture of these aromatic cages allows the high-affinity binding of various osmostress protectants with fully methylated head-groups via cation–π interactions (Schiefner et al., 2004a; Mahadevi and Sastry, 2013) regardless whether the head-group contains a nitrogen, sulfur, arsenic, selenium, or tellurium atom (Schiefner et al., 2004a; Broy et al., 2015; Hoffmann et al., 2018). The design principle of the architecture of aromatic cages present in substrate-binding proteins is evolutionarily conserved between *Archaea* and both Gram-negative and Gram-positive *Bacteria*. It is apparent from the physicochemical properties of compatible solutes (Capp et al., 2009) that evolution has found a common solution allowing the efficient and specific binding of types of organic osmolytes by proteins that are otherwise preferentially excluded from protein surfaces (Bolen and Baskakov, 2001; Ignatova and Gierasch, 2006; Street et al., 2006; Stadmiller et al., 2017). In addition, small differences in the architecture or composition of aromatic cages can significantly affect the efficiency of ligand binding or the substrate specificity of solute receptor proteins functioning with ABC transporters and of the *C. glutamicum* BetP transporter (Smits et al., 2008; Du et al., 2011; Perez et al., 2011; Pittelkow et al., 2011; Tschapek et al., 2011). Hence, an aromatic cage would be well-suited to accommodate the inducers choline (for GbsR) (Nau-Wagner et al., 2012) and choline and glycine betaine (for OpuAR) (Table 1) to trigger the release of these repressor proteins from their operator sequences.

The side chains of the six aromatic amino acids (all Phe residues) (Table 1) presumably form an aromatic cage in the *B. subtilis* GbsR protein (Figures 1A,C). Two of these six aromatic residues are replaced in the *B. infantis* OpuAR protein with either an Asn (N) or Arg (R) residue (Figure 8A, Table 1), possibly providing an explanation for the reduced affinity of OpuAR for its ligands in comparison with GbsR (Table 1). The purified OpuAR protein binds its inducer glycine betaine with a *K*_d_ value of 301 ± 24 μM, yet the addition of 5 μM glycine betaine to the growth medium already triggers a notable effect on *opuAA-treA* transcription (Figure 7). The comparison of these *in vitro* and *in vivo* generated datasets thus indicates that osmotically stressed *Bacillus* cells need to accumulate the inducers(s) of the OpuAR repressor protein above a certain cytoplasmic threshold level to trigger enhanced *opuA* expression in order to provide osmostress protection through OpuA-mediated compatible solute import (Figure 5B).

Mutational analysis of amino acid residues forming aromatic cages in various substrate-binding proteins has demonstrated that the replacement of these aromatic amino acids with charged, polar, or neutral amino acids has a strong negative effect on ligand binding. There are even cases where the substitution of an aromatic amino acid by another amino acid impairs ligand binding (Schiefner et al., 2004a; Smits et al., 2008; Pittelkow et al., 2011; Tschapek et al., 2011). The negative effects on ligand binding by substrate-binding proteins with mutationally altered aromatic cages could, in a worst-case scenario, be critically explained through indirect effects on protein structure. By relying on information gleaned from the mutational analysis of substrate-binding proteins, we rationally designed two amino acid substitutions in the putative aromatic cage of OpuAR with the intention to improve ligand binding, and this is precisely what we observed (Table 1). We consider it unlikely that indirect effects on protein structure caused by the Y^94^F and R^100^F double amino acid substitutions could somehow lead to an enhanced affinity of OpuAR for its inducers choline and glycine betaine.

All things appropriately considered, we take the data summarized in Table 1 as compelling evidence that the proposed aromatic cage in GbsR- and OpuAR-type regulatory proteins (Figures 1A,C, 8A) constitutes indeed the inducer-binding site of these repressor proteins. It is hoped that among the large number of GbsR-/OpuAR-type proteins identified in this study (Figure 2 and Supplementary Figure S5), candidates suitable for crystallographic analysis can be found in order to reveal the true three-dimensional structure of these physiologically important group of regulatory proteins.

## Author Contributions

EB conceived and supervised the study. SR, BW, and CA conducted the experiments and interpreted their results. BW designed all the figures, and BW and EB jointly wrote the manuscript.

## Conflict of Interest Statement

The authors declare that the research was conducted in the absence of any commercial or financial relationships that could be construed as a potential conflict of interest.

## Footnotes

1 http://img.jgi.doe.gov/cgi-bin/pub/main.cgi

2 http://mafft.cbrc.jp/alignment/server/

3 https://itol.embl.de/

4 http:://swissmodel.expasy.org

5 http://www.pymol.org

6 http://img.jgi.doegov/cgi-bin/pub/main.cgi/
