# Supplementary material for: The GbsR Family of Transcriptional Regulators: Functional Characterization of the OpuAR Repressor
Source: Front Microbiol. 2018 Oct 24;9:2536. doi: 10.3389/fmicb.2018.02536 (PMC6207618; doi:10.3389/fmicb.2018.02536)
Supplement: Supplementary file 1 [file Data_Sheet_1.PDF]

The GbsR family of transcriptional regulators: functional characterization of the OpuAR repressor

Stefanie Ronzheimer[1¶], Bianca Warmbold[1¶], Christian Arnhold[1], and Erhard Bremer[1,2]*

[1]Laboratory for Microbiology, Department of Biology, Philipps-University Marburg, Marburg, Germany

[2]LOEWE-Center for Synthetic Microbiology, Philipps-University Marburg, Marburg, Germany

[¶]These authors have contributed equally to this work

For correspondence please contact:

Dr. Erhard Bremer, Philipps-University Marburg, Dept. of Biology, Laboratory for Microbiology, Karl-von-Frisch-Str. 8, D-35032 Marburg, Germany. Phone: (+49)-6421-2821529. Fax: (+49)-6421-2828979. E-Mail: bremer@staff.uni-marburg.de

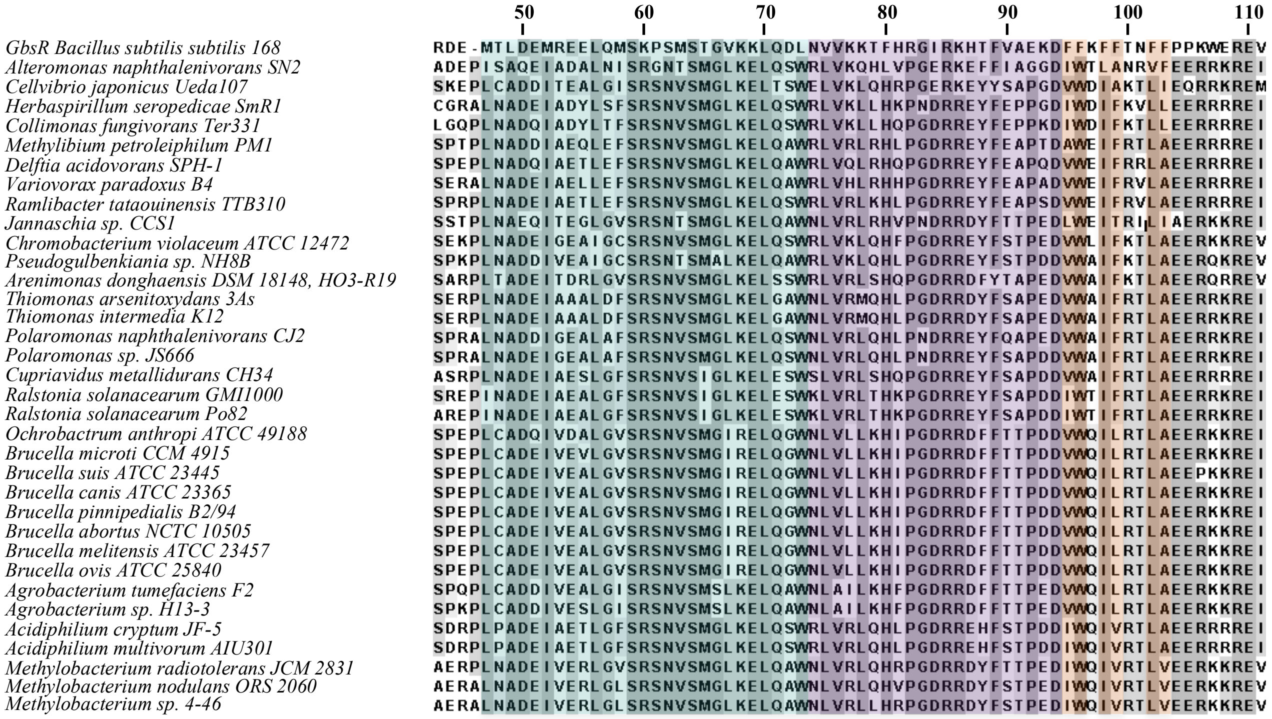

Supplementary Figure S1│ Amino acid sequence alignment of CydE-type proteins. The amino acid sequences of 34 CydE-type proteins (Xia et al., 2018) were compared to the amino acid sequence of the *B. subtilis* GbsR protein (Nau-Wagner et al., 2012) by an alignment using the MAFFT server (Katoh et al., 2017). Depicted are only the N-terminal domains of these proteins, containing the amino acids corresponding to the winged helix-turn-helix DNA-binding motif (lite blue), the inter-domain linker (purple) and of the likely inducer-binding site (reddish) of GbsR. Highly conserved amino acids are shaded in grey

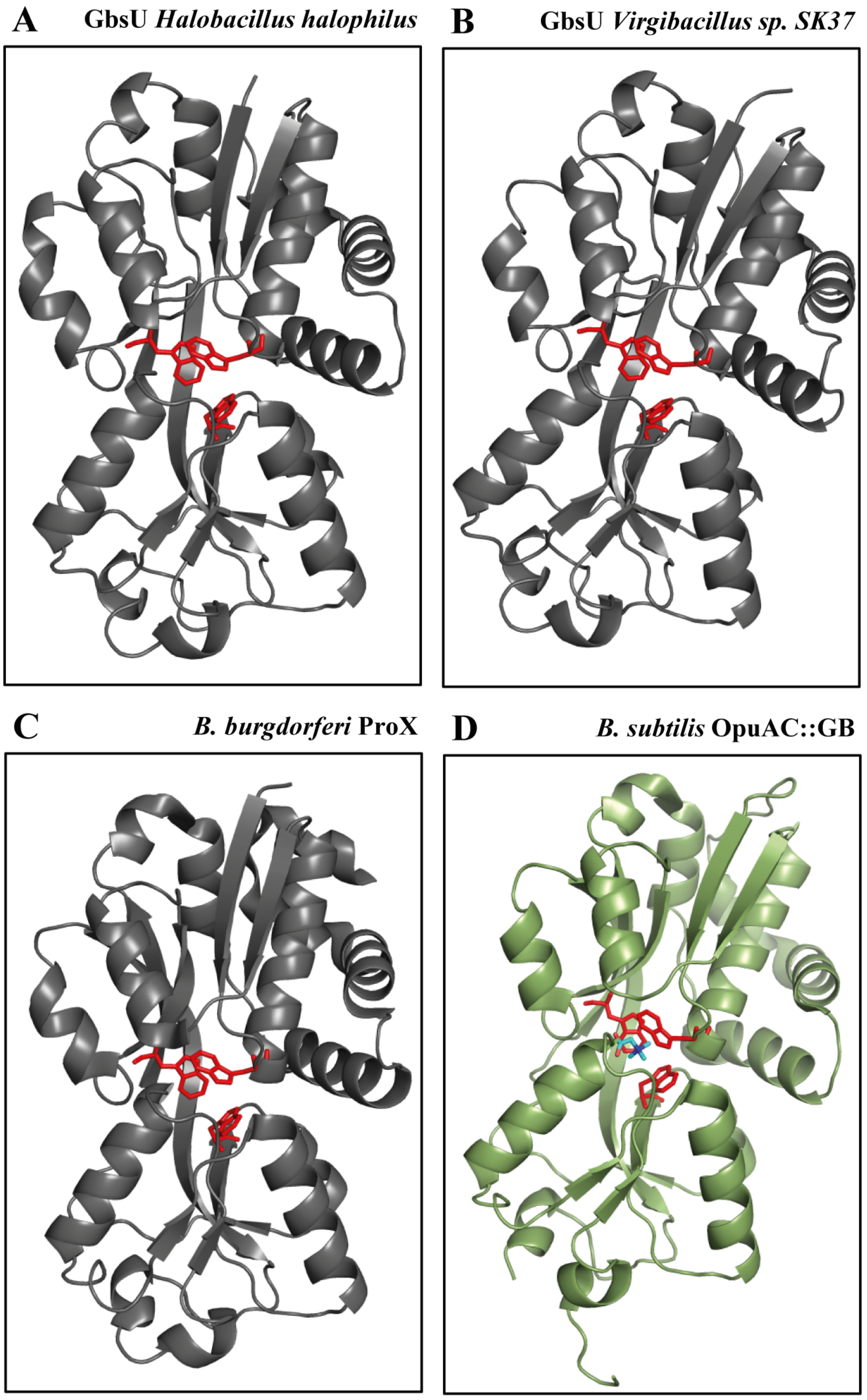

Supplementary Figure S2│In *silico* models of the GbsU substrate-binding proteins. Depicted are the substrate-binding proteins encoded within the putative glycine betaine synthesis gene clusters of (**A**) *Halobacillus halophilus* DSM 2266 and (**B**) *Virgibacillus sp.* SK37 based on the crystal structure of **(C)** ProX of *Borrelia burgdorferi* [PDB entry 3TMG; (SSGCID, Gardberg, A., Fox, D., Staker, B., Stewart, L.; *to be published.*)]. All of these substrate-binding proteins show a ligand-binding site similar to the OpuAC protein of *B. subtilis*. Its crystal structure in complex with the ligand glycine betaine [PDB entry 2B4L (Horn et al., 2006)] is depicted in panel **D**. Aromatic amino acids likely to be involved in ligand-binding of the *H. halophilus* DSM 2266 and *Virgibacillus sp.* SK37 GbsU proteins are highlighted in red. Glycine betaine, the substrate of the OpuAC protein of *B. subtilis* (Horn et al., 2006) is shown in blue.

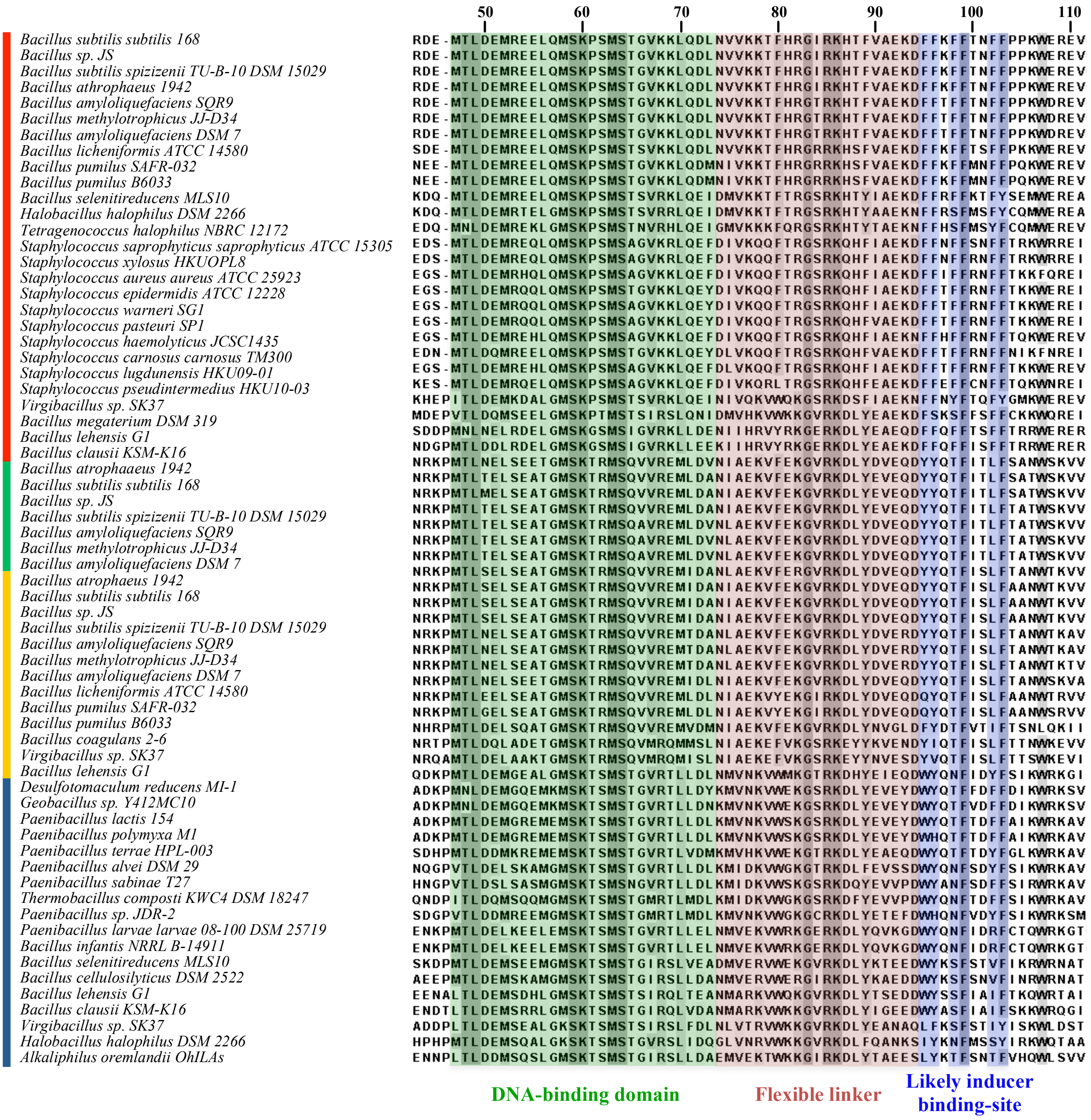

Supplementary Figure S3 │ Amino acid sequence alignment of GbsR-type proteins associated with osmotic stress. The amino acid sequences of 27 GbsR-type proteins associated with glycine betaine synthesis (red), 18 OpuAR-type proteins (blue), 7 GbsR-type proteins associated with OpuB-type transporters (green) and 13 GbsR-type proteins associated with OpuC-type ABC transporters (yellow) were aligned using the MAFFT server (Katoh et al., 2017). Depicted are the N-terminal domains of these proteins. The amino acids corresponding to the winged helix-turn-helix DNA-binding motif (green), the inter-domain linker (reddish), and of the likely inducer-binding site (blue) are highlighted. Highly conserved amino acids are shaded in grey.

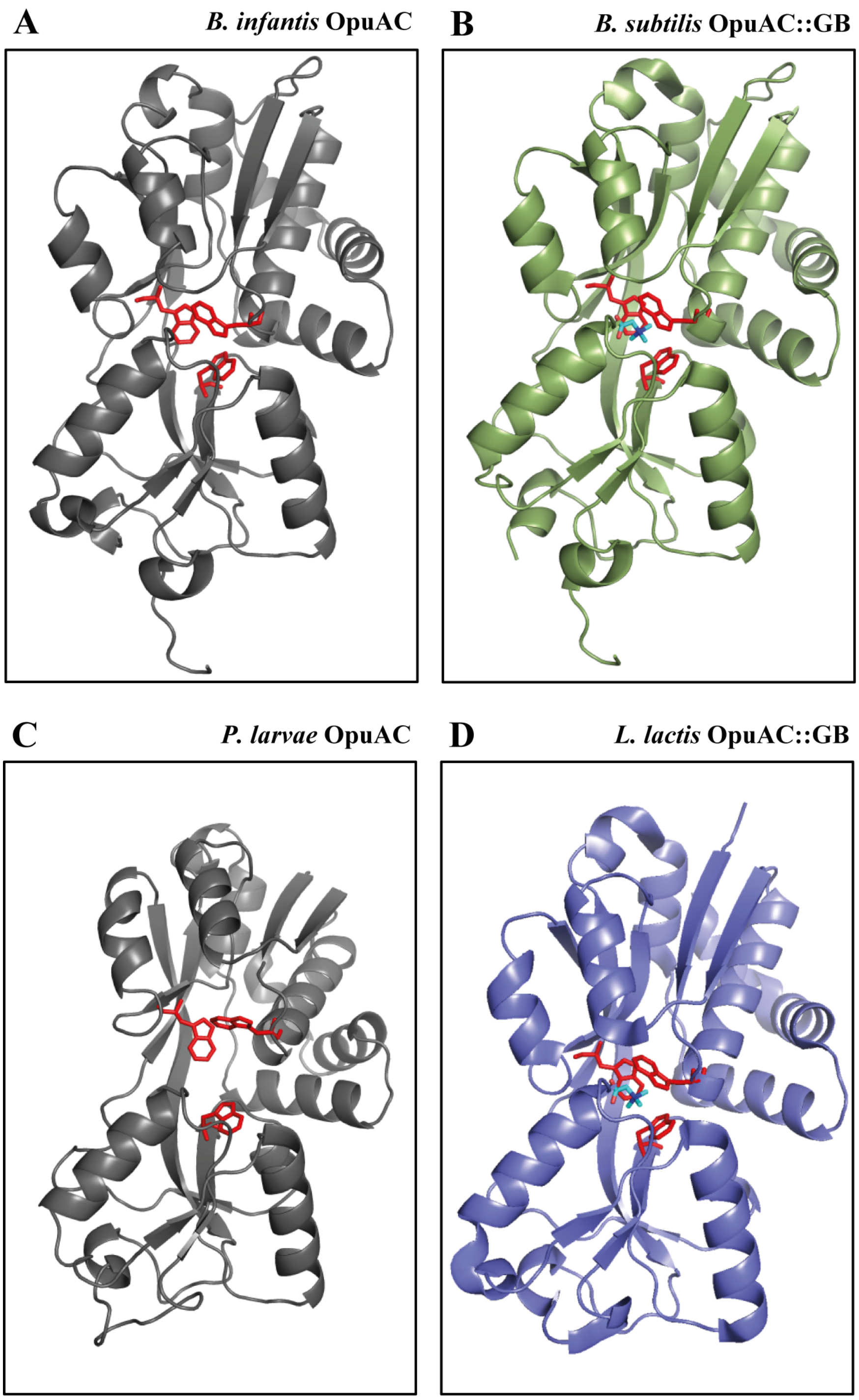

Supplementary Figure S4 │Structural arrangements of the OpuAC substrate-binding proteins. Shown are the *in silico* derived models of the OpuAC proteins of **(A)** *B. infantis* based on the crystal structure of **(B)** the *B. subtilis* OpuAC protein in complex with its ligand glycine betaine [PDB entry 2B4L (Horn et al., 2006)] and, **(C)** the modeled substrate-binding domain of the fused OpuABC protein of *P. larvae larvae* 08-100 DSM 25719 based on the crystallographic data on the (**D**) *L. lactis* OpuAC complexed with glycine betaine [PDB entry 3L6H (Wolters et al., 2010)]. Aromatic amino acids involved in the binding of glycine betaine are highlighted in red, whereas the ligand glycine betaine is depicted as blue sticks.

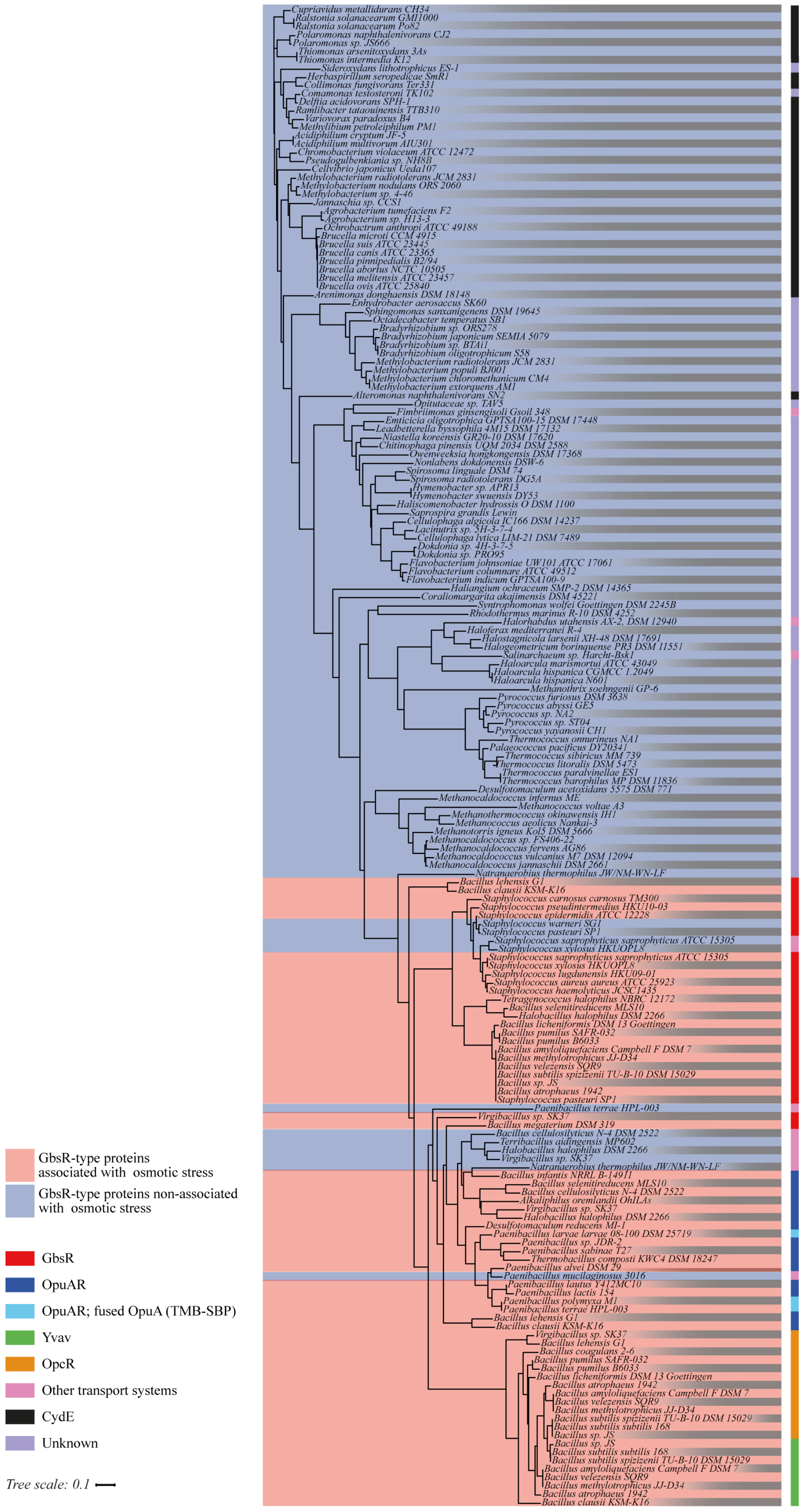
Cupriavidus metallidurans CH34
Ralstonia solanacearum GMI1000
Ralstonia solanacearum Po82
Polaromonas naphthalenivorans CJ2
Polaromonas sp. JS666
Thiomonas arsenitoxydans 3As
Thiomonas intermedia K12
Sideroxydans lithotrophicus ES-1
Herbaspirillum seropedicae SmR1
Collimonas fungivorans Ter331
Comamonas testosteroni TK102
Delftia acidovorans SPH-1
Ramlibacter tataouinensis TTB310
Variovorax paradoxus B4
Methylibium petroleiphilum PM1
Acidiphilium cryptum JF-5
Acidiphilium multivorum AIU301
Chromobacterium violaceum ATCC 12472
Pseudogulbenkiania sp. NH8B
Cellvibrio japonicus Ueda107
Methylobacterium radiotolerans JCM 2831
Methylobacterium nodulans ORS 2060
Methylobacterium sp. 4-46
Jannaschia sp. CCS1
Agrobacterium tumefaciens F2
Agrobacterium sp. H13-3
Ochrobactrum anthropi ATCC 49188
Brucella microti CCM 4915
Brucella suis ATCC 23445
Brucella canis ATCC 23365
Brucella pinnipedialis B2/94
Brucella abortus NCTC 10505
Brucella melitensis ATCC 23457
Brucella ovis ATCC 25840
Arenimonas donghaensis DSM 18148
Enhydrobacter aerosaccus SK60
Sphingomonas sanxanigenens DSM 19645
Octadecabacter temperatus SB1
Bradyrhizobium sp. ORS278
Bradyrhizobium japonicum SEMIA 5079
Bradyrhizobium sp. BTAi1
Bradyrhizobium oligotrophicum S58
Methylobacterium radiotolerans JCM 2831
Methylobacterium populi BJ001
Methylobacterium chloromethanicum CM4
Methylobacterium extorquens AM1
Alteromonas naphthalenivorans SN2
Opitutaceae sp. TAV5
Fimbriimonas ginsengisoli Gsoil 348
Emticicia oligotrophica GPTSA100-15 DSM 17448
Leadbetterella byssophila 4M15 DSM 17132
Niastella koreensis GR20-10 DSM 17620
Chitinophaga pinensis UQM 2034 DSM 2588
Owenweeksia hongkongensis DSM 17368
Nonlabens dokdonensis DSW-6
Spirosoma linguale DSM 74
Spirosoma radiotolerans DG5A
Hymenobacter sp. APR13
Hymenobacter swuensis DY53
Haliscomenobacter hydrossis O DSM 1100
Saprospira grandis Lewin
Cellulophaga algicola IC166 DSM 14237
Lacinutrix sp. 5H-3-7-4
Cellulophaga lytica LIM-21 DSM 7489
Dokdonia sp. 4H-3-7-5
Dokdonia sp. PRO95
Flavobacterium johnsoniae UW101 ATCC 17061
Flavobacterium columnare ATCC 49512
Flavobacterium indicum GPTSA100-9
Haliangium ochraceum SMP-2 DSM 14365
Coraliomargarita akajimensis DSM 45221
Syntrophomonas wolfei Goettingen DSM 2245B
Rhodothermus marinus R-10 DSM 4252
Halorhabdus utahensis AX-2, DSM 12940
Haloferax mediterranei R-4
Halostagnicola larsenii XH-48 DSM 17691
Halogeometricum borinquense PR3 DSM 11551
Salinarchaeum sp. Harcht-Bsk1
Haloarcula marismortui ATCC 43049
Haloarcula hispanica CGMCC 1.2049
Haloarcula hispanica N601
Methanothrix soehngenii GP-6
Pyrococcus furiosus DSM 3638
Pyrococcus abyssi GE5
Pyrococcus sp. NA2
Pyrococcus sp. ST04
Pyrococcus yayanosii CH1
Thermococcus onnurineus NA1
Palaeococcus pacificus DY20341
Thermococcus sibiricus MM 739
Thermococcus litoralis DSM 5473
Thermococcus paralvinellae ES1
Thermococcus barophilus MP DSM 11836
Desulfotomaculum acetoxidans 5575 DSM 771
Methanocaldococcus infernus ME
Methanococcus voltae A3
Methanothermococcus okinawensis IH1
Methanococcus aeolicus Nankai-3
Methanotorris igneus Kol5 DSM 5666
Methanocaldococcus sp. FS406-22
Methanocaldococcus fervens AG86
Methanocaldococcus vulcanius M7 DSM 12094
Methanocaldococcus jannaschii DSM 2661
Natranaerobius thermophilus JW/NM-WN-LF
Bacillus lehensis G1
Bacillus clausii KSM-K16
Staphylococcus carnosus carnosus TM300
Staphylococcus pseudintermedius HKU10-03
Staphylococcus epidermidis ATCC 12228
Staphylococcus warneri SG1
Staphylococcus pasteuri SP1
Staphylococcus saprophyticus saprophyticus ATCC 15305
Staphylococcus xylosus HKUOPL8
Staphylococcus saprophyticus saprophyticus ATCC 15305
Staphylococcus xylosus HKUOPL8
Staphylococcus lugdunensis HKU09-01
Staphylococcus aureus aureus ATCC 25923
Staphylococcus haemolyticus JCSC1435
Tetragenococcus halophilus NBRC 12172
Bacillus selenitireducens MLS10
Halobacillus halophilus DSM 2266
Bacillus licheniformis DSM 13 Goettingen
Bacillus pumilus SAFR-032
Bacillus pumilus B6033
Bacillus amyloliquefaciens Campbell F DSM 7
Bacillus methylotrophicus JJ-D34
Bacillus velezensis SQR9
Bacillus subtilis spizizenii TU-B-10 DSM 15029
Bacillus sp. JS
Bacillus atrophaeus 1942
Staphylococcus pasteuri SP1
Paenibacillus terrae HPL-003
Virgibacillus sp. SK37
Bacillus megaterium DSM 319
Bacillus cellulosilyticus N-4 DSM 2522
Terribacillus aidingensis MP602
Halobacillus halophilus DSM 2266
Virgibacillus sp. SK37
Natranaerobius thermophilus JW/NM-WN-LF
Bacillus infantis NRRL B-14911
Bacillus selenitireducens MLS10
Bacillus cellulosilyticus N-4 DSM 2522
Alkaliphilus oremlandii OhILAs
Virgibacillus sp. SK37
Halobacillus halophilus DSM 2266
Desulfotomaculum reducens MI-1
Paenibacillus larvae larvae 08-100 DSM 25719
Paenibacillus sp. JDR-2
Paenibacillus sabinae T27
Thermobacillus composti KWC4 DSM 18247
Paenibacillus alvei DSM 29
Paenibacillus mucilaginosus 3016
Paenibacillus lautus Y412MC10
Paenibacillus lactis 154
Paenibacillus polymyxa M1
Paenibacillus terrae HPL-003
Bacillus lehensis G1
Bacillus clausii KSM-K16
Virgibacillus sp. SK37
Bacillus lehensis G1
Bacillus coagulans 2-6
Bacillus pumilus SAFR-032
Bacillus pumilus B6033
Bacillus licheniformis DSM 13 Goettingen
Bacillus atrophaeus 1942
Bacillus amyloliquefaciens Campbell F DSM 7
Bacillus velezensis SQR9
Bacillus methylotrophicus JJ-D34
Bacillus subtilis spizizenii TU-B-10 DSM 15029
Bacillus subtilis subtilis 168
Bacillus sp. JS
Bacillus sp. JS
Bacillus subtilis subtilis 168
Bacillus subtilis spizizenii TU-B-10 DSM 15029
Bacillus amyloliquefaciens Campbell F DSM 7
Bacillus velezensis SQR9
Bacillus methylotrophicus JJ-D34
Bacillus atrophaeus 1942
Bacillus clausii KSM-K16
GbsR-type proteins associated with osmotic stress
GbsR-type proteins non-associated with osmotic stress
GbsR
OpuAR
OpuAR; fused OpuA (TMB-SBP)
Yvav
OpcR
Other transport systems
CydE
Unknown
Tree scale: 0.1

Supplementary Figure S5│ Clade analysis of GbsR-type proteins among *Bacteria* and *Archaea*. 179 GbsR-type proteins were identified by bioinformatics. Information on fully sequenced microbial genomes were obtained from the IMG/M Web-server and homologs of the GbsR protein of *B. subtilis* JH642 (Nau-Wagner et al., 2012) were searched for via the JGI Web-server (Chen et al., 2017) using the BLAST-P algorithm (Altschul et al., 1990). The retrieved amino acid sequences were aligned using the MAFFT Web-server (Katoh et al., 2017) with bootstrapping setting (100 bootstraps) proved by the Web-server to analyze their phylogenetic relationship. The protein-homology tree was visualized using the iTOL web-tool (Letunic and Bork, 2016). GbsR homologs were grouped according to their gene neighborhood as depicted by colored boxes. If the *gbsR*-type gene was encoded in proximity of genes associated with glycine betaine synthesis or uptake systems for osmostress protectants it was classified as a member of the sub-group of GbsR-type proteins associated with osmotic stress (highlighted in red). Otherwise the GbsR homologs were assigned as non-associated with osmotic stress (highlighted in blue).

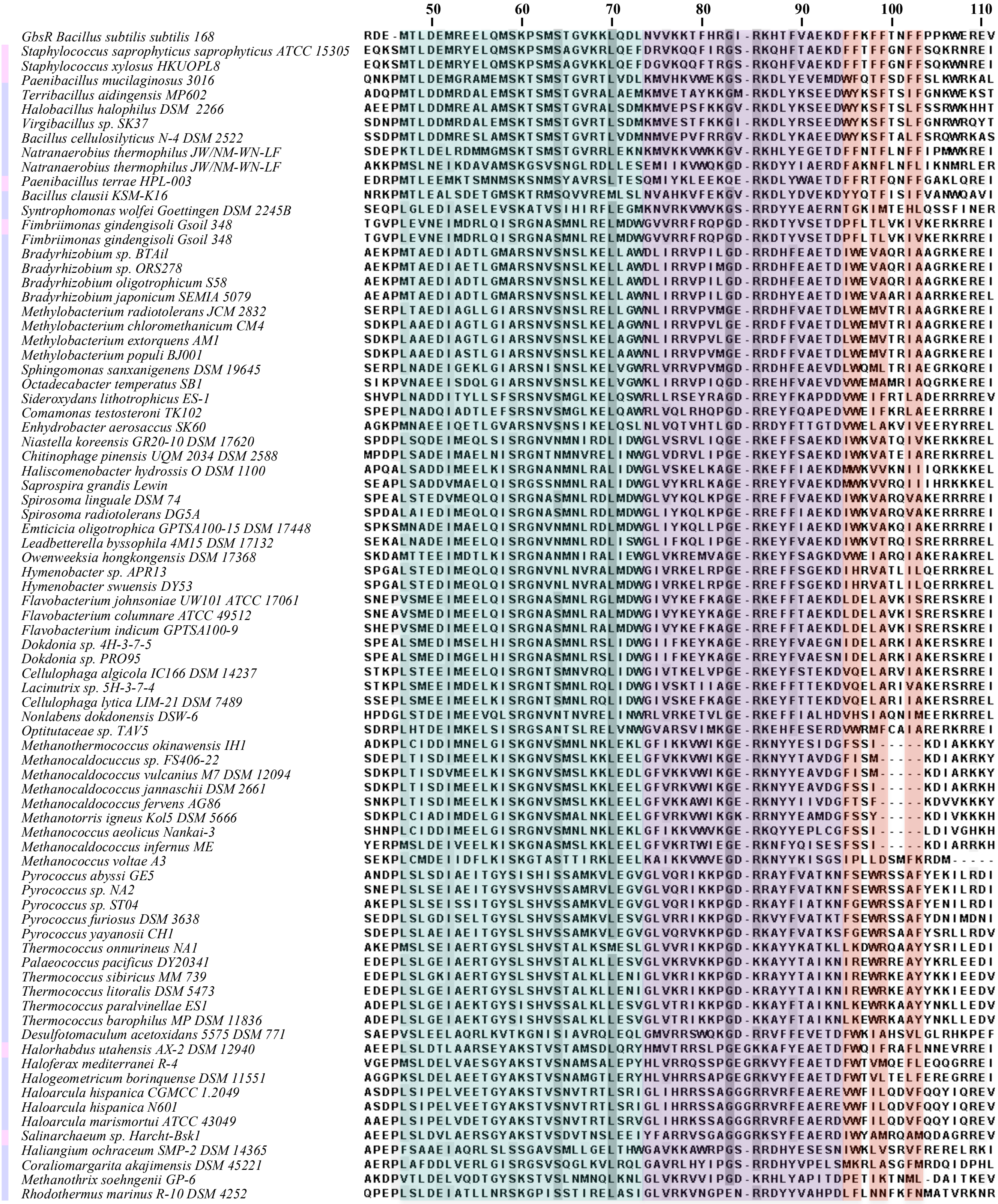

Supplementary Figure S6 │ Amino acid sequence alignment of GbsR-type proteins non-associated with osmotic stress. The amino acid sequences of 7 GbsR homologs associated with various transport systems (pink) and 73 GbsR-type proteins, encoded in a gene neighborhood which does not allow the assignment of a particular function (pale purple) were aligned using the MAFFT server (Katoh et al., 2017). Depicted are only the N-terminal domains of these proteins. The amino acids corresponding to the winged helix-turn-helix DNA-binding motif (lite blue), the inter-domain linker (purple) and of the likely inducer-binding site (reddish) of GbsR are highlighted. Highly conserved amino acids are shaded in grey.

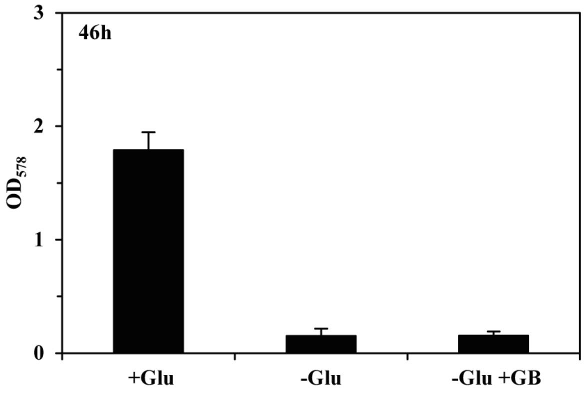

Supplementary Figure S7 │*B. infantis* cannot use glycine betaine as carbon source. Cultures of *B. infantis* NRRL B-14911were grown in basal medium supplemented with either glucose (27.7 mM final concentration) or glycine betaine (33.24 mM final concentration) as the sole carbon source. Growth yields were measured after 46 hours of incubation at 37 °C. Cultures without the addition of a carbon source served as control.

Supplementary Table S1 │ *Bacillus subtilis* strains used in this study

Strain	Relevant genotype	Source or reference
JH642	*trpC2 pheA1*	J. Hoch; BGSC* 1A96
TMB118	*Δ(opuA::tet)3 Δ(opuC::spc)3 Δ(opuD::neo)2 Δ(opuB::erm)3*	(Teichmann et al., 2017)
GNB37	*Δ(treA::erm)2*	(Nau-Wagner et al., 2012)
GNB40	*Δ(gbsR::neo)1 Δ(treA::erm)2*	(Nau-Wagner et al., 2012)
CAB1	*Δ(opuA::tet)3 Δ(opuC::spc)3 Δ(opuD::neo)2 Δ(opuB::erm)3 amyE::pX*	This study
CAB2	*Δ(opuA::tet)3 Δ(opuC::spc)3 Δ(opuD::neo)2 Δ(opuB::erm)3 amyE::opuAR-opuA*$_{B.i}$	This study
STHB04	Δ*(treA::erm)2* Δ*(gbsR::neo)1* Δ*(yvaV::tet)2*	This study
STHB05	Δ*(treA::erm)2* Δ*(gbsR::neo)1* Δ*(yvaV::tet)2* Δ*(opcR::zeo)2*	This study
STHB06	Δ*(treA::erm)2* Δ*(gbsR::neo)1* Δ*(opcR::zeo)2*	This study
STHB07	Δ*(treA::erm)2* Δ*(yvaV::tet)2* Δ*(opcR::zeo)2*	This study
STHB08	Δ*(treA::erm)2* Δ*(opcR::zeo)2*	This study
STHB10	Δ*(treA::erm)2* Δ*(gbsR::neo)1* Δ*(yvaV::tet)2* Δ*(opcR::zeo)2 [amyE::Φ(opuAA*$_{B.i}$*′-treA); opuAR*$^{+}$*]1*	This study
STHB11	Δ*(treA::erm)2* Δ*(gbsR::neo)1* Δ*(yvaV::tet)2* Δ*(opcR::zeo)2 [amyE::Φ(opuAA′-treA)]1*	This study
STHB65	Δ*(treA::erm)2* Δ*(gbsR::neo)1* Δ*(yvaV::tet)2 [amyE::Φ(opuAA′*$_{B.i}$*-treA)]1*	This study
STHB66	Δ*(treA::erm)2* Δ*(gbsR::neo)1* Δ*(opcR::zeo)2 [amyE::Φ(opuAA′*$_{B.i}$*-treA)]1*	This study
STHB67	Δ*(treA::erm)2* Δ*(yvaV::tet)2* Δ*(opcR::zeo)2 [amyE::Φ(opuAA′*$_{B.i}$*-treA)]1*	This study

*BGSC: Bacillus Genetic Stock Center (Columbus, OH, USA).

Supplementary Table S2 │ Primers used in this study

Primer name	Primer sequence (5´-3´)
OpcR Knout P1	ATAAATTCTTCAACAAACTCATTTGCCGG
OpcR Knout Zeo P2	CCATATCAAGATAACTTCGTATAATGTATGTTGAAGGCATTCCAAACGTATGCATATTTT
OpcR Knout Zeo P3	CCATATCAAGATAACTTCGTATAATGTATGTTGAAGGCATTCCAAACGTATGCATATTTT
OpcR Knout P4	GTAAAGCAATACTCGTCTGCTTTTGTTTTA
OpcR Knout Zeo P5	AAAATATGCATACGTTTGGAATGCCTTCAACATACATTATACGAAGTTATCTTGATATGG
OpcR Knout Zeo P6	TATTTTAGAGAGCTGCATTCTTTTGTTTTCTAATGTATGCTATACGAAGTTATTCAGTCC
YvaV Knout P1	GATAAATTCCTCAACAAATTCGTCTGCC
Yvav Knout Tet P2	CCGTAATGCTATGTTAGCATTACTCTTTTCCATGTTTTCCGCGATTCTTTCTATAAAATG
YvaV Kneu2 Tet P3	AAATTGTTATCCGCTCACAATTCCACACAACATATTTGAGAGCGAAGACATTTTTAAATATGTG
YvaV Knout P4	CAGTGAAATAAACCGGTAAATCTAGGTCTC
YvaV Knout Tet P5	CATTTTATAGAAAGAATCGCGGAAAACATGGAAAAGAGTAATGCTAACATAGCATTACGG
YvaV Kneu2 Tet P6	CACATATTTAAAAATGTCTTCGCTCTCAAATATGTTGTGTGGAATTGTGAGCGGATAACAATTT
OpuAR fwd	ATGGTAGGTCTCAAATGCATAACGAAGAATTGGTCGCACAG
OpuAR rev	ATGGTAGGTCTCAGCGCTCTCTTCCTTTTTTTCATCCTCAGAC
OpuAR_Box1_for	CCAGGTCAAAGGGGATTGGTTCCAGAATTTCATCGATCGCTTTTGTACG
OpuAR_Box1_rev	CGTACAAAAGCGATCGATGAAATTCTGGAACCAATCCCCTTTGACCTGG
OpuAR_Box3_for	GGTACCAGAATTTCATCGATTTCTTTTGTACGCAATGGAGAAAAGG
OpuAR_Box3_rev	CCTTTTCTCCATTGCGTACAAAAGAAATCGATGAAATTCTGGTACC
OpuAR_Box1+3_for	CCAGGTCAAAGGGGATTGGTTCCAGAATTTCATCGATTTCTTTTGTACG
OpuAR_Box1+3_rev	CGTACAAAAGAAATCGATGAAATTCTGGAACCAATCCCCTTTGACCTGG
OpuAR_N98Fn_for	GTACAAAAGCGATCGATGAAAAACTGGTACCAATCCCCTTTGAC
OpuAR_N98Fn_rev	TCAAAGGGGATTGGTACCAGTTTTTCATCGATCGCTTTTGTAC
OpuAR_W95F_for	GATTTATACCAGGTCAAAGGGGATTTCTACCAGAATTTCATCGATCG
OpuAR_W95F_rev	CGATCGATGAAATTCTGGTAGAAATCCCCTTTGACCTGGTATAAATC
CA3-opuARA fwd	AAAGGATCCGCATCTACTCTTCCTTTTTTTC
CA3-opuARA rev	AAAGGATCCCATTAATCTAAGTCTTCGATTG
OpuAR treA Frag1/4 for	AAACCCGGGGCACCTTTTTGGAGAGCATCTAC
OpuAR treA Frag2 for	AAACCCGGGGAACTCCGTATAAATGCATGTTTC
OpuAR treA Frag1/2 rev	AAAGGATCCCTGAAGCAGCTTCGATTGTGAC

Supplementary Table S3 | Plasmids used in this study

Plasmid	Description	Resistance	Reference
pASK-IBA3plus	Expression plasmid for *E. coli* with a AHT-inducible *tet*-promoter and a C-terminal *Strep*-tag II	*bla*	IBA (Göttingen, Germany)
pX	Integration vector for *B. subtilis* *amyE::cat::amyE*	*bla, cat*	(Kim et al., 1996)
pJMB1	*amyE::treA*	*bla, cat*	(Hoffmann et al., 2013)
pDG1515	Tetracycline resistance cassette	*tet*	(Guerout-Fleury et al., 1995)
p7Z6	Zeocin resistance cassette	*zeo*	(Yan et al., 2008)
pCA-*opuARA*	*opuA* operon and *opuAR* gene of *B. infantis* with native promoters cloned into pX-*amyE*-site	*bla, cat*	This study
pSTH33	*amyE::*Φ(*opuAA*$_{B.i}$′*-treA*)*; opuAR*$^{+}$	*bla, cat*	This study
pSTH34	*amyE::*Φ(*opuAA*$_{B.i}$′*-treA*)	*bla, cat*	This study
pMP_AR1	*B. infantis opuAR* gene cloned into pASK-IBA3plus	*bla*	This study
pSTH49	Site directed mutagenesis of *B. infantis opuAR* in pMP_AR1: *opuAR* [W^{93}F]	*bla*	This study
pSTH50	Site directed mutagenesis of *B. infantis opuAR* in pMP_AR1: *opuAR* [N^{96}F]	*bla*	This study
pOpuAR_Y94F	Site directed mutagenesis of *B. infantis opuAR* in pMP_AR1: *opuAR* [Y^{94}F]	*bla*	This study
pOpuAR_R100F	Site directed mutagenesis of *opuAR* in pMP_AR1: *B. infantis opuAR* [R^{100}F]	*bla*	This study
pOpuAR_Y94F_R100F	Site directed mutagenesis of *B. infantis opuAR* in pMP_AR1: *opuAR* [Y^{94}F/R^{100}F]	*bla*	This study

REFERENCES

Altschul, S.F., Gish, W., Miller, W., Myers, E.W., and Lipman, D.J. (1990). Basic local alignment search tool. *J. Mol. Biol.* 215**,** 403-410.

Chen, I.A., Markowitz, V.M., Chu, K., Palaniappan, K., Szeto, E., Pillay, M., et al. (2017). IMG/M: integrated genome and metagenome comparative data analysis system. *Nucleic Acids Res.* 45**,** D507-D516. doi: 10.1093/nar/gkw929.

Guerout-Fleury, A.M., Shazand, K., Frandsen, N., and Stragier, P. (1995). Antibiotic-resistance cassettes for *Bacillus subtilis*. *Gene* 167**,** 335-336.

Hoffmann, T., Wensing, A., Brosius, M., Steil, L., Völker, U., and Bremer, E. (2013). Osmotic control of *opuA* expression in *Bacillus subtilis* and its modulation in response to intracellular glycine betaine and proline pools. *J. Bacteriol.* 195**,** 510-522. doi: 10.1128/JB.01505-12.

Horn, C., Sohn-Bösser, L., Breed, J., Welte, W., Schmitt, L., and Bremer, E. (2006). Molecular determinants for substrate specificity of the ligand-binding protein OpuAC from *Bacillus subtilis* for the compatible solutes glycine betaine and proline betaine. *J. Mol. Biol.* 357**,** 592-606. doi: 10.1016/j.jmb.2005.12.085.

Katoh, K., Rozewicki, J., and Yamada, K.D. (2017). MAFFT online service: multiple sequence alignment, interactive sequence choice and visualization. *Brief Bioinform***,** 1-7. doi: 10.1093/bib/bbx108.

Kim, L., Mogk, A., and Schumann, W. (1996). A xylose-inducible *Bacillus subtilis* integration vector and its application. *Gene* 181**,** 71-76.

Letunic, I., and Bork, P. (2016). Interactive tree of life (iTOL) v3: an online tool for the display and annotation of phylogenetic and other trees. *Nucleic Acids Res.* 44**,** W242-W245. doi: 10.1093/nar/gkw290.

Nau-Wagner, G., Opper, D., Rolbetzki, A., Boch, J., Kempf, B., Hoffmann, T., et al. (2012). Genetic control of osmoadaptive glycine betaine synthesis in *Bacillus subtilis* through the choline-sensing and glycine betaine-responsive GbsR repressor. *J. Bacteriol.* 194**,** 2703-2714. doi: JB.06642-11 10.1128/JB.06642-11.

Teichmann, L., Chen, C., Hoffmann, T., Smits, S.H.J., Schmitt, L., and Bremer, E. (2017). From substrate specificity to promiscuity: hybrid ABC transporters for osmoprotectants. *Mol. Microbiol.* 104**,** 761-780. doi: 10.1111/mmi.13660.

Wolters, J.C., Berntsson, R.P., Gul, N., Karasawa, A., Thunnissen, A.M., Slotboom, D.J., et al. (2010). Ligand binding and crystal structures of the substrate-binding domain of the ABC transporter OpuA. *PloS one* 5**,** e10361. doi: 10.1371/journal.pone.0010361.

Xia, X., Wu, S., Li, L., Xu, B., and Wang, G. (2018). The cytochrome *bd* complex Is essential for chromate and sulfide resistance and is regulated by a GbsR-type regulator, CydE, in *Alishewanella* sp. WH16-1. *Front. Microbiol.* 9**,** 1849. doi: 10.3389/fmicb.2018.01849.

Yan, X., Yu, H.J., Hong, Q., and Li, S.P. (2008). Cre/lox system and PCR-based genome engineering in *Bacillus subtilis*. *Appl. Environ. Microbiol.* 74**,** 5556-5562. doi: AEM.01156-08 10.1128/AEM.01156-08.
